# Activation of Tripartite Motif Containing 63 Expression by Transcription Factor EB and Transcription Factor Binding to Immunoglobulin Heavy Chain Enhancer 3 Is Regulated by Protein Kinase D and Class IIa Histone Deacetylases

**DOI:** 10.3389/fphys.2020.550506

**Published:** 2021-01-13

**Authors:** Cristina Pablo Tortola, Britta Fielitz, Yi Li, Julia Rüdebusch, Friedrich C. Luft, Jens Fielitz

**Affiliations:** ^1^Experimental and Clinical Research Center (ECRC), Max Delbrück Center (MDC) for Molecular Medicine in the Helmholtz Association, Charité-Universitätsmedizin Berlin, Berlin, Germany; ^2^Department of Internal Medicine B, Cardiology, University Medicine Greifswald, Greifswald, Germany; ^3^DZHK (German Center for Cardiovascular Research), Partner Site Greifswald, Greifswald, Germany

**Keywords:** muscle atrophy, protein kinase D, HDAC = histone deacetylase, transcription factor EB, TFE3, muscle ring finger protein 1

## Abstract

**Rationale:**

The ubiquitin–proteasome system (UPS) is responsible for skeletal muscle atrophy. We showed earlier that the transcription factor EB (TFEB) plays a role by increasing E3 ubiquitin ligase muscle really interesting new gene-finger 1(MuRF1)/*tripartite motif-containing 63* (*TRIM63*) expression. MuRF 1 ubiquitinates structural proteins and mediates their UPS-dependent degradation. We now investigated how TFEB-mediated *TRIM63* expression is regulated.

**Objective:**

Because protein kinase D1 (PKD1), histone deacetylase 5 (HDAC5), and TFEB belong to respective families with close structural, regulatory, and functional properties, we hypothesized that these families comprise a network regulating *TRIM63* expression.

**Methods and Results:**

We found that TFEB and transcription factor for immunoglobulin heavy-chain enhancer 3 (TFE3) activate *TRIM63* expression. The class IIa HDACs HDAC4, HDAC5, and HDAC7 inhibited this activity. Furthermore, we could map the HDAC5 and TFE3 physical interaction. PKD1, PKD2, and PKD3 reversed the inhibitory effect of all tested class IIa HDACs toward TFEB and TFE3. PKD1 mediated nuclear export of all HDACs and lifted TFEB and TFE3 repression. We also mapped the PKD2 and HDAC5 interaction. We found that the inhibitory effect of PKD1 and PKD2 toward HDAC4, HDAC5, and HDAC7 was mediated by their phosphorylation and 14-3-3 mediated nuclear export.

**Conclusion:**

TFEB and TFE3 activate *TRIM63* expression. Both transcription factors are controlled by HDAC4, HDAC5, HDAC7, and all PKD-family members. We propose that the multilevel PKD/HDAC/TFEB/TFE3 network tightly controls *TRIM63* expression.

## Introduction

Muscle mass is mainly regulated by an equilibrium of protein synthesis and degradation. Both muscle atrophy and hypertrophy occur physiologically and under disease conditions (Hershko and Ciechanover, 1998; Ciechanover, 2006; Schmidt et al., 2014; Wollersheim et al., 2014). The ubiquitin–proteasome system (UPS) is the principal protein-degrading system in muscle and largely responsible for the degradation of contractile proteins (Medina et al., 1995; Wing et al., 1995; Voisin et al., 1996; Ciechanover, 2006; Witt et al., 2008). Substrate protein ubiquitination is mediated by E3 ubiquitin-ligases that are specificity assuring and rate-limiting enzymes within the UPS (Hershko and Ciechanover, 1998; Ciechanover, 2006). Polyubiquitinated target proteins are then degraded by the 26S proteasome (Ciechanover, 2006). The UPS is activated during muscle atrophy leading to declining of structural and contractile proteins, most notably myosin heavy chain (MHC) (Fielitz et al., 2007), resulting in a reduction of muscle mass and function. The E3 ligase, muscle really interesting new gene-finger 1 (MuRF1), was identified as a major atrogene and is restricted to skeletal muscle and the heart (Bodine et al., 2001a). MuRF1 was shown to mediate ubiquitination and UPS-dependent degradation of structural proteins, such as alpha-actin, troponin I, troponin T, telethonin, titin, nebulin, the nebulin-related-anchoring protein, myosin light chain 2, myotilin, and T-cap (Kedar et al., 2004; Witt et al., 2005; Polge et al., 2011, 2018). Importantly, MuRF1 is also involved in muscular energy metabolism by degradation of non-structural proteins, such as muscle-type creatine kinase, glucocorticoid modulatory element binding protein-1, and 3-hydroxyisobutyrate dehydrogenase in striated muscles (McElhinny et al., 2002; Koyama et al., 2008). Numerous studies showed that the *tripartite motif-containing 63* (*TRIM63*) gene expression, encoding MuRF1, is increased rapidly and strongly during various physiological and pathological atrophy conditions (Bodine et al., 2001a). The multitude of its target proteins, together with its strong transcriptional regulation, highlights the importance of MuRF1 in muscle homeostasis. To evaluate the transcriptional control of *TRIM63*, we performed a complementary DNA (cDNA)-expression screen to identify regulators of *TRIM63* expression and identified the basic helix-loop-helix leucine zipper (bHLH-LZ) transcription factor EB (TFEB) as a novel *TRIM63* regulator (Du Bois et al., 2015). We found that TFEB binds to specific, well-conserved, enhancer box (E-box) DNA motifs in the *TRIM63* promoter that are close to its transcription start site. We reported that TFEB activity was inhibited via interaction with the class IIa histone deacetylase (HDAC) 5. We also showed that inhibition of TFEB by HDAC5 was attenuated by the stress-dependent serine/threonine kinase protein kinase D (PKD) 1, which interacted with HDAC5 and mediated its phosphorylation and 14-3-3 chaperone mediated nuclear export. This PKD1/HDAC5/TFEB axis controlled the expression of *TRIM63* and was found to be important in angiotensin II-induced myocyte atrophy *in vitro* and muscle atrophy *in vivo* (Du Bois et al., 2015).

Recent reports, however, indicated that this pathway might not be as specific as we believed. For example, TFEB belongs to the microphthalmia/transcription factor E (MiT/TFE) family of bHLH-LZ transcription factors (TFs), which also includes TFE3, TFEC, and microphthalmia-associated transcription factor (MiTF). All MiT/TFEs recognize a unique E-box motif within the proximal promoters of lysosomal and autophagy genes (Sardiello et al., 2009; Palmieri et al., 2011; Martina et al., 2014) and regulate cellular catabolism and nutrient-dependent lysosomal response (Settembre et al., 2011; Slade and Pulinilkunnil, 2017). Importantly, TFEB and TFE3 were found to have partially redundant functions and to regulate overlapping sets of genes (Pastore et al., 2016). However, whether or not TFE3 or MiTF can activate *TRIM63* expression is not known. In addition, the class IIa HDACs (HDAC4, HDAC5, HDAC7, and HDAC9) are highly expressed in the heart and skeletal muscle, and their interaction with the myocyte enhancer factor 2 (MEF2) TF decreases the expression of MEF2 target genes (Lu et al., 2000a,b; McKinsey et al., 2000; Zhang et al., 2002). If other class IIa HDACs inhibit the activity of TFEB or TFE3 is also unclear. Finally, PKD1 belongs to a family of calmodulin-calcium-dependent serine-threonine kinases (termed PKD1, PKD2, and PKD3). These kinases are important in cell growth, differentiation, migration, and apoptosis (Rozengurt et al., 2005). The close structural relationship of the PKD family members suggested that they might phosphorylate overlapping targets. Indeed, all three family members PKD1, PKD2, and PKD3 were shown to phosphorylate HDAC5 (Huynh and McKinsey, 2006). However, any effect on TFEB or TFE3-mediated *TRIM63* expression is uncertain. Because PKD1, HDAC5, and TFEB belong to respective families with close structural, regulatory, and functional properties, we hypothesized that other family members could also be involved in the regulation of *TRIM63* expression.

## Materials and Methods

### Cell Culture, Complementary DNA Expression Plasmids, and Luciferase Reporter Assays

Cell culture experiments of murine myoblasts (C2C12 cells, ATCC, United States) were performed as previously described (Langhans et al., 2014; Huang et al., 2017; Zhu et al., 2017). Briefly, myoblasts were cultured in the growth medium [Dulbecco’s modified Eagle’s medium (1 g/l glucose, Merck, Germany), 10% fetal bovine serum (Biochrom GmbH, Germany), supplemented with penicillin and streptomycin (Merck, Germany)]. For Western blot analyses, immunostaining and co-immunoprecipitation experiments, the C2C12 cells were cultivated on six-well plates, coverslips and 10 cm dishes, respectively. COS-7 cells were cultured in standard cell culture conditions using Dulbecco’s modified Eagle medium (4.5 g/l glucose, L-glutamine, 10% fetal bovine serum, and penicillin–streptomycin). Cells were seeded in 24-well plates in triplicates and transfected with cDNA expression plasmids, vector control, and pGL3-*TRIM63*_Luc reporter construct, as indicated, using FuGENE6^®^ (Promega) transfection reagent according to the manufacturer’s protocol. To control transfection efficacy, 25 ng of pCMV lacZ (Clontech) was co-transfected in each sample. Cell pellets were lysed in 200 μl Luciferase Cell Culture Lysis Reagent (Promega). Fifty microliters of lysate was used for quantification of luciferase activity and β-galactosidase in a luminometer (FLUOstar Optima, BMG-Labtech). The Luciferase Assay System (Promega) was used to quantify the expression of the reporter gene constructs. Luciferase activity was normalized to fluorescence measured with the FluoReporter^®^ lacZ/Galactosidase Quantification Kit (Invitrogen). The cDNA expression plasmids [pcDNA3.1-TFEB-C-MYC, pcDNA3.1-TFEB-N- FLAG, pcDNA3.1-PKD1-CA-N-MYC, pcDNA3.1-HDAC4-MYC, pcDNA3.1-HDAC5-MYC, pcDNA3.1-HDAC7-MYC, pcDNA3.1-C-MYC, pcDNA3.1-C-MYC, and pcDNA3.1-N-FLAG (Vega et al., 2004; Fielitz et al., 2008; Kim et al., 2008; Du Bois et al., 2015), HDAC5-deletion mutants (Song et al., 2006; Du Bois et al., 2015), and further constructs (pGL3-*TRIM63*_Luc, pGL3-*TRIM63*_mut_E-box1_Luc, pGL3-*TRIM63*_mut_E-box2_Luc, pGL3-*TRIM63*_mut_E-box3_Luc, and pCMV lacZ) (Fielitz et al., 2008; Kim et al., 2008; Du Bois et al., 2015)] were recently published. The cDNA expression plasmids for human HDAC1 (#13820) (Emiliani et al., 1998), human HDAC3 (#13819) (Emiliani et al., 1998), and human calcium/calmodulin-dependent protein kinase type IV (CamK IV, #126422) (Xie and Black, 2001) were purchased from Addgene, United States. For the generation of the cDNA expression vectors pcDNA3.1-TFE3-N-FLAG, pcDNA3.1-MiTF-N-FLAG, pcDNA3.1-PKD2-CA-N-MYC, pcDNA3.1-PKD3-CA-N-MYC, pcDNA3.1-PKD2-CA-N-FLAG, and pcDNA3.1-PKD3-CA-N-FLAG, expressing murine TFE3, MiTF, constitutive active PKD2 or PKD3 with an N-terminal FLAG tag or an N-terminal MYC tag, respectively, mouse cDNA was PCR amplified with the primers shown in Online Table 1 using Advantage HD Polymerase (Takara) and cloned into pcDNA-3.1-N-MYC or pcDNA-3.1-N-FLAG (both Invitrogen) using the restriction enzymes indicated and T4 DNA ligase (both New England Biolabs) according to the manufacturer’s protocol. All constructs were verified by sequencing. The mammalian two-hybrid assay was performed as recently published (Chang et al., 2005; Du Bois et al., 2015).

**TABLE 1:** Primers used for generation of cDNA expression plasmids.

**Primer**	**Sequence**
TFE3 pcDNA3.1-N-FLAG forward (*Cla*I)	5′-CCA TCG ATT CTC ATG CAG CCG AGC-3′
TFE3 pcDNA3.1-N-FLAG reverse (*Xba*I)	5′-GCT CTA GAT CAG GAC TCC TCT TCC ATG CT-3′
MiTF pcDNA3.1-N-FLAG forward (*Cla*I)	5′-CCA TCG ATC AGT CCG AAT CGG GAA TC-3′
MiTF pcDNA3.1-N-FLAG reverse (*Xba*I)	5′-GCT CTA GAC TAA CAC GCA TGC TCC GTT TC-3′
PKD2 pcDNA3.1-CA-N-MYC forward (*Cla*I)	5′-CCA TCG ATG CCG CCG CCC CCT CCC ATC CCG-3′
PKD2 pcDNA3.1-CA-N-MYC reverse (*Eco*RI)	5′-GAA TTC TCA GAG GAT GCT GAT GCG CTC AGC-3′
PKD2 pcDNA3.1-CA-N-FLAG forward (*Eco*RI)	5′-CGG AAT TCG CCG CCG CCC CCT CCC ATC C-3′
PKD2 pcDNA3.1-CA-N-FLAG reverse (*Not*I)	5′-ATA GTT TAG CGG CCG CCA GAG GAT GCT GAT GCG CTC-3′
PKD3 pcDNA3.1-CA-N-MYC forward (*Cla*I)	5′-CCA TCG ATT CTG CAA ATA ATT CCC CTC CA-3′
PKD3 pcDNA3.1-CA-N-MYC reverse (*Eco*RI)	5′-GAA TTC CTA AGG ATC CTC CTC CAT GT-3′
PKD3 pcDNA3.1-CA-N-FLAG forward (*Xho*I)	5′-CCG CTC GAG CTA AGG ATC CTC CTC CAT GTC G-3′
PKD3 pcDNA3.1-CA-N-FLAG reverse (*Eco*RI)	5′-CGG AAT TCT CTG CAA ATA ATT CCC CTC C-3′

### Immunofluorescence

For immunostaining, C2C12 myoblasts were cultured in 8 chamber polystyrene vessel tissue culture treated glass slides (BD Biosciences, United States) and fixed with 4% paraformaldehyde/PBS, permeabilized with 0.2% Triton-X-100/PBS, and blocked with 2% goat serum/PBS. The following primary antibodies were used: anti-TFEB antibody (Biolegend, United States, 1:100), anti-TFE3 antibody (Abnova, 1:50), anti-PKD1 antibody (Abnova, 1:80), anti-PKD3 antibody (Sigma-Aldrich, United States 1:100), anti-HDAC4 antibody (Cell Signaling United Kingdom, 1:100), anti-HDAC5 antibody (Cell Signaling United Kingdom, 1:100), and anti-HDAC7 antibody (Cell Signaling United Kingdom, 1:100). Alexa fluor 488 Goat Anti-Mouse IgG (H + L) (Life Technologies, United States, 1:1,000) and Alexa fluor 555 Goat Anti-Rabbit IgG (H + L) (Life Technologies, United States, 1:1,000) were used as secondary antibodies. 4′,6-diamidino-2-phenylindole, dihydrochloride (300 mM, Thermo Fisher Scientific, United States) was used to stain for nuclei. Immunostained cells were embedded in ProLong^TM^ Gold antifade mountant (Life Technologies, United States). Images were acquired with a Zeiss LSM 700 confocal microscope and processed with ZEN 2009 (Zeiss) and Fiji software.

COS-7 cells were plated in six-well plates on sterile coverslips. At the experimental endpoint, cells were PBS-washed and fixed in 3.7% formaldehyde for 10 min at room temperature. Permeabilization and blocking of cells were carried out in a single step with 0.3% Triton X-100, 0.5% goat serum (Abcam) in PBS for 1 h at room temperature. Coverslips were incubated with the specific primary antibody at 4°C overnight. Secondary antibodies conjugated with Alexa Fluor^®^ 488 or Alexa Fluor^®^ 555 were diluted in PBS and incubated for 2 h at room temperature. 4′,6-diamidino-2-phenylindole, dihydrochloride (300 mM, Thermo Fisher Scientific, United States) was used to stain for nuclei. Immunostained cells were embedded in ProLong^TM^ Gold antifade mountant (Invitrogen, United States). Images were acquired with a Zeiss LSM 700 confocal microscope and processed with ZEN 2009 (Zeiss) and Fiji software.

### Protein Extraction and Western Blot Analysis

Western blot analysis was performed as recently published (Schmidt et al., 2014; Lodka et al., 2016; Huang et al., 2017; Zhu et al., 2017). Shortly, cells were lysed in ice-cold extraction buffer [10-mM Tris-hydrochloride, pH 7.5, 140-mM sodium chloride, 1-mM ethylenediaminetetraacetic acid, 25% glycerol, 0.5% sodium dodecyl sulfate (SDS), 0.5% Non-idet P-40, 0.1-mM dithiothreitol, 0.5-mM phenylmethylsulfonyl fluoride, and 100 ng/ml complete ethylenediaminetetraacetic acid-free protease inhibitor cocktail, ROCHE] and then cleared by centrifugation (4°C, 15 min, 12,000 × *g*). The Bio-Rad Protein Assay was used to quantitate protein concentration in the supernatant. Isolated proteins were frozen and stored at −80°C until usage. For Western blot analysis, 20 μg protein was separated by 10% SDS polyacrylamide gel electrophoresis and blotted onto polyvinylidene fluoride or nitrocellulose membranes (GE Healthcare, United Kingdom). Membranes were incubated with specific primary antibodies: anti-glyceraldehyde-3-phosphate dehydrogenase (GAPDH, clone 6C5, Millipore, United States, 1:10,000), anti-HDAC4 antibody (Cell Signaling, United Kingdom, 1:1,000), anti-HDAC5 antibody (Cell Signaling, United Kingdom, 1:1,000), anti-HDAC7 antibody (Cell Signaling, United Kingdom, 1:1,000), anti-phospho-HDAC4 (Ser246)/HDAC5 (Ser259)/HDAC7 (Ser155) (Cell Signaling United Kingdom, 1:1,000), anti-phospho-HDAC4 (Ser632)/HDAC5 (Ser661)/HDAC7 (Ser486) (Cell Signaling United Kingdom, 1:1,000), anti-DYKDDDDK (FLAG-tag, Cell Signaling United Kingdom, 1:1,000), and anti-MYC (Millipore, 1:500) and secondary horseradish peroxidase-conjugated antibodies: anti-mouse IgG (Cell Signaling, United Kingdom, 1:3,000), anti-rabbit IgG (Cell Signaling, United Kingdom, 1:20,000), and anti-goat IgG (Abcam, United Kingdom, 1:3,000). The signal^TM^ was visualized with Super Signal West Pico Chemiluminescent Substrate (Thermo Fisher Scientific, United States).

### Co-immunoprecipitation

Transfected cells were washed with ice-cold PBS and resuspended in lysis buffer (50 mM potassium phosphate, 150 mM sodium chloride, 0.5% Triton X-100, pH 7.4). Lysates were cleared by centrifugation at 10,000 × *g* for 10 min at 4°C. For co-immunoprecipitation, supernatants were incubated with 30 μl of prewashed anti-FLAG M2 agarose (Sigma, A2220) for 2 h at 4°C. Immunoprecipitated proteins were eluted from agarose by 5 min boiling at 95°C in Laemmli sample buffer (125-mM Tris-hydrochloride, 10% glycerol, 10% SDS, 130-mM dithiothreitol) and analyzed by Western blot.

### Statistics

All experiments were performed independently and at least three times using biological triplicates each and technical duplicates for each biological replicate. Data are shown as mean ± SEM. For all comparisons, one-way analyses of variance (ANOVAs) followed by a pairwise Student’s *t*-test for independent groups were performed using GraphPad Prism 8.3.0 (GraphPad Software, Inc.) and Excel 2016 (Microsoft) software. *P*-values of less than 0.05 were considered statistically significant (^∗^*P* < 0.05; ^∗∗^*P* < 0.01; ^∗∗∗^*P* < 0.001). Adobe Illustrator, version 16.0.0, was used for illustrations.

## Results

### Class IIa Histone Deacetylases 4, 5, and 7 Inhibit Transcription Factor EB-Mediated *Tripartite Motif-Containing 63* Expression

**FIGURE 1:**
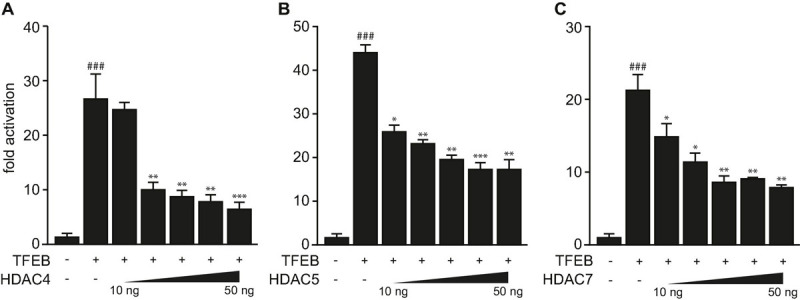
Class IIa histone deacetylases 4, 5, and 7 inhibit TFEB-induced TRIM63 expression. **(A–C)** Luciferase assays performed with cell extracts of COS-7 cells transfected with Hs_*TRIM63*-Luc (-543 bp), FLAG-TFEB (TFEB) and increasing amounts of **(A)** MYC-HDAC4 (HDAC4), **(B)** MYC-HDAC5 (HDAC5), or **(C)** MYC-HDAC7 (HDAC7) as indicated or control (-) plasmid. Luciferase activity was normalized to expression of CMV-LacZ and expressed as fold increase. Data are represented as mean ± SEM. One-way ANOVA *P* < 0.0001 for **(A–C)**; ^###^*P* < 0.005 vs. control transfection with pcDNA3.1; *P < 0.05, **P < 0.01, ***P < 0.005 vs. transfection with TFEB only.

Previously, we showed that TFEB increases the expression of human *TRIM63* by binding to specific E-box elements close to the transcription start site in its promoter (Du Bois et al., 2015). We showed that TFEB transcriptional activity was attenuated by HDAC5 that physically interacted and colocalized with TFEB in myocytes *in vitro* (Du Bois et al., 2015). Because HDAC4 and HDAC5 collectively inhibit the activity of the bHLH TF myogenin toward *TRIM63* expression (Moresi et al., 2010), we hypothesized that other class IIa HDACs reduce the activity of TFEB on the *TRIM63* promoter as well. Using luciferase assays, we found that HDAC4 (Figure 1A), HDAC5 (Figure 1B), and HDAC7 (Figure 1C) inhibit TFEB-induced activity of the human *TRIM63* luciferase reporter in a dose-dependent manner. These data indicate that TFEB-induced *TRIM63* expression is inhibited by not only HDAC5 but also HDAC4 and HDAC7.

### Protein Kinase D Family Members Attenuate Histone Deacetylase-Mediated Inhibition of Transcription Factor EB-Induced *Tripartite Motif-Containing 63* Expression

**FIGURE 2:**
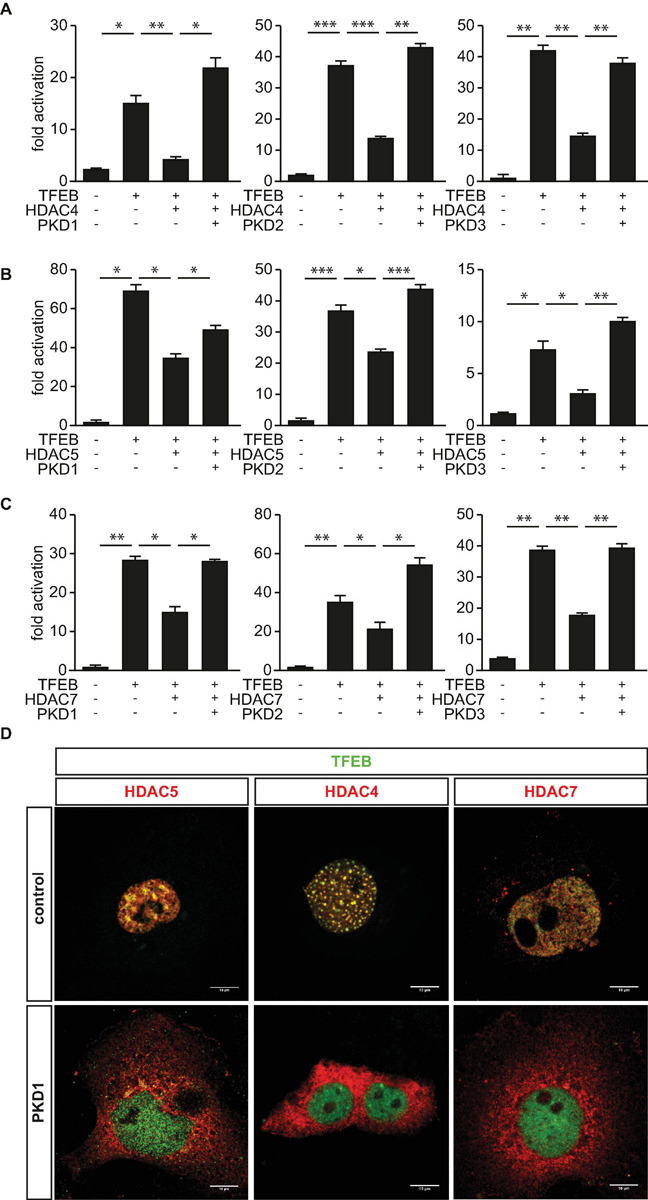
Protein kinase D family members attenuate HDAC-mediated inhibition of TFEB-induced *TRIM63* expression. **(A–C)**, COS-7 cells were transfected with expression plasmids encoding FLAG-TFEB, **(A)** HDAC4-MYC, **(B)** HDAC5-MYC, or **(C)** HDAC7-MYC, or constitutively active (ca) PKD1 (left panel), caPKD2 (middle panel), and caPKD3 (right panel) proteins, as indicated, together with the Hs_*TRIM63*_Luc reporter construct (-543 bp). Values were normalized to expression of CMV-LacZ and calculated as the fold increase in luciferase/CMV-LacZ ratio compared with the reporter alone. Data are represented as mean ± SEM. One-way ANOVA *P* < 0.0001 for **(A–C)**; *P < 0.05; **P < 0.01; ***P < 0.005. *n* = 5. **(D)** COS-7 cells were transfected with FLAG-TFEB and HDAC4-MYC, HDAC5-MYC, and HDAC7-MYC together with a pcDNA3.1 control vector or caPKD1, as indicated. Immunostaining was performed with anti-FLAG (green) and anti-MYC (red) antibodies. Scale bars, 10 μm.

Earlier, we reported that PKD1 associates with phosphorylates and facilitates 14-3-3-mediated nuclear export of HDAC5, which relives inhibition of TFEB and thereby increases *TRIM63* expression (Du Bois et al., 2015). Because the three PKD-family members PKD1, PKD2, and PKD3 share a high degree of similarity in their functional domains (Avkiran et al., 2008) and were shown to phosphorylate HDAC5 (Huynh and McKinsey, 2006), we hypothesized that the PKD-family regulates TFEB activity by inactivating HDAC4, HDAC5, and HDAC7 as well. To investigate the effect on HDAC-mediated inhibition of TFEB, we performed luciferase assays. As already shown, TFEB-induced *TRIM63* activity was inhibited by HDAC4 (Figure 2A), HDAC5 (Figure 2B), and HDAC7 (Figure 2C). In contrast, the class I HDACs 1 and 3 did not decrease TFEB-induced *TRIM63* activity (Supplementary Figure 1A). When we cotransfected PKD1 (Figures 2A–C, left panels), PKD2 (Figures 2A–C, middle panels), or PKD3 (Figures 2A–C, right panels), the repressive effects of all three HDACs were relieved. CamK IV, which was used as a positive control, also relieved the repressive effects of HDAC4, HDAC5, and HDAC7 in the same assay (Supplementary Figure 2A). Our data indicate that the PKD family converges on HDAC4, HDAC5, and HDAC7 to control *TRIM63* expression. To investigate if the observed effects of the PKD family onto HDAC-mediated inhibition of TFEB are due to nuclear export of HDAC4 and HDAC7, we performed immunocytochemistry experiments. As expected, we observed colocalization of HDAC5 (Figure 2D, left panel) and also HDAC4 (Figure 2D, middle panel) and HDAC7 (Figure 2D, right panel) with TFEB in the nucleus. This colocalization is a possible molecular basis for class IIa HDAC-mediated TFEB inhibition. When we cotransfected with PKD1, HDAC4, HDAC5, and HDAC7 translocated to the cytoplasm, whereas TFEB remained in the nucleus (Figure 2D).

**FIGURE 3:**
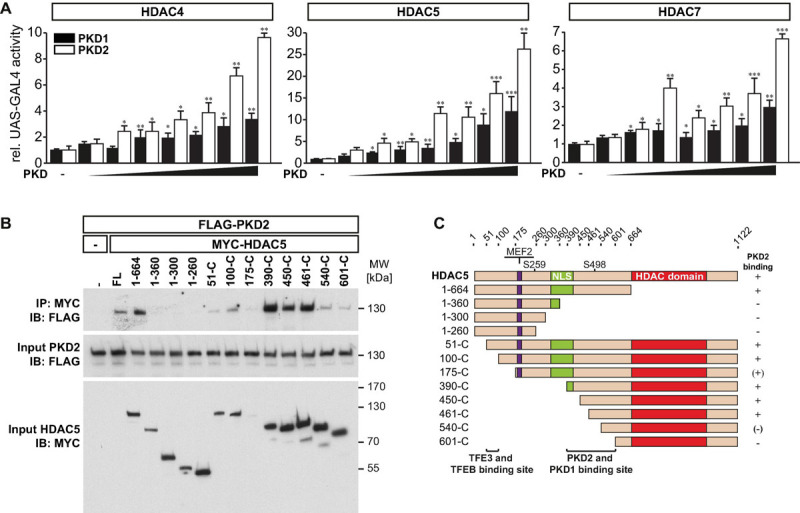
PKD1 and PKD2 mediate phosphorylation and 14-3-3 binding of class IIa HDACs. **(A)** COS-7 cells were transfected with upstream activator sequence (UAS)-luciferase and expression plasmids encoding GAL4 fused with the wild-type (WT) HDAC4 (left panel), HDAC5 (middle panel), or HDAC7 (right panel) N-terminal extension together with 14-3-3-VP16 and increasing amounts of expression plasmids of constitutively active (ca) PKD1 or caPKD2 (from 6.25 to 400 ng), as indicated. Values were normalized to expression of CMV-LacZ and calculated as fold increase. Data are represented as mean ± SEM. One-way ANOVA *P* < 0.0001 for all; *P < 0.05; **P < 0.01; ***P < 0.005. *n* = 3. **(B)**, Co-immunoprecipitation (Co-IP) assay with lysates from COS-7 cells expressing FLAG-PKD2 and deletion mutants of MYC-HDAC5, as indicated. HDAC5 fusion proteins were immunoprecipitated (IP) with anti-MYC antibodies, and PKD2 fusion proteins were detected with an antibody directed against FLAG. Input proteins were detected by Western blot (immunoblot, IB) with antibodies directed against the FLAG- or MYC-tag. *n* = 3. FL indicates full length. **(C)** Scheme of PKD2 binding site in HDAC5.

Phosphoserines 246, 467, and 632 in HDAC4 (Backs et al., 2006), 259 and 498 in HDAC5 (Zhang et al., 2002), and 155, 181, 321, and 449 in HDAC7 (Dequiedt et al., 2005) serve as binding sites for the chaperone protein 14-3-3 and are known PKD1 phosphorylation sites. Previously, we confirmed that PKD1 binds to HDAC5 and phosphorylates its 14-3-3 consensus sites. To investigate if PKD1 has a similar effect on HDAC4 and HDAC7, we performed a mammalian-two-hybrid assay using GAL4 upstream activator sequence (UAS)-luciferase, as published recently (Chang et al., 2005; Du Bois et al., 2015). This assay was also used to investigate if PKD2 phosphorylates HDAC4, HDAC5, and HDAC7 as well. In this assay, the N-terminus of HDAC4, HDAC5, or HDAC7 is fused to the GAL4 DNA-binding domain, and 14-3-3 is fused to the VP16 transactivation domain. Under normal growth conditions, these HDACs are not phosphorylated in COS-7 cells. Thus, GAL4-HDAC4, GAL4-HDAC5, or GAL4-HDAC7 cannot interact with 14-3-3-VP16, and the GAL4-dependent UAS-luciferase cannot be activated (Figure 3A). Expression plasmids encoding these fusion proteins, together with UAS-luciferase, were transfected into COS-7 cells together with increasing amounts of PKD1 or PKD2 expression plasmids. PKD1 and PKD2 increased UAS-luciferase activity in a dose-dependent manner. These data indicate that PKD1 and PKD2 create phospho-14-3-3 recognition motifs in HDAC4, HDAC5, and HDAC7, which recruit 14-3-3 proteins that mediate their nuclear export.

We had identified amino acids 360-601 as PKD1 binding region in HDAC5 (Du Bois et al., 2015). To test if PKD2 and HDAC5 physically interact as well, we performed co-immunoprecipitation experiments with PKD2 and wild-type and deletion mutants of HDAC5. We found that PKD2 interacted avidly with HDAC5 and that amino acids 360–601 in HDAC5 were responsible for this interaction (Figures 3B,C).

To investigate if PKD1, PKD2, and PKD3 phosphorylate endogenous HDAC4, HDAC5, and HDAC7 in myocytes, we transfected the respective PKD expression plasmids into C2C12 cells and performed Western blot analyses on proteins isolated from these cells using anti-phospho-HDAC4 (Ser246)/HDAC5 (Ser259)/HDAC7 (Ser155), anti-phospho-HDAC4 (Ser632)/HDAC5 (Ser661)/HDAC7 (Ser486), anti-HDAC4, anti-HDAC5, anti-HDAC7, and anti-glyceraldehyde-3-phosphate dehydrogenase antibodies. PKD1, PKD2, and PKD3 phosphorylated all three endogenous class IIa HDACs in C2C12 cells (Supplementary Figure 3A). We used the pan-PKD inhibitor CID 2011756 to investigate the specificity of PKD1-mediated HDAC4-, HDAC5-, and HDAC7-phosphorylation in myocytes. C2C12 myocytes were transfected with PKD1 or pcDNA and treated with CID 2011756 (50 μM) or vehicle (dimethyl sulfoxide) for 6 h. Western blot analyses of protein lysates showed that CID 2011756 attenuated PKD1-mediated phosphorylation of all three endogenous class IIa HDACs in myocytes (Supplementary Figure 3B). In summary, these data indicate that PKD1, PKD2, and PKD3 attenuate the inhibitory effect of HDAC4, HDAC5, and HDAC7 onto TFEB-induced *TRIM63* expression by phosphorylation and 14-3-3 mediated nuclear export of these HDACs.

### Transcription Factor Binding to Immunoglobulin Heavy-Chain Enhancer 3 but Not Microphthalmia-Associated Transcription Factor Increase *Tripartite Motif-Containing 63* Expression

**FIGURE 4:**
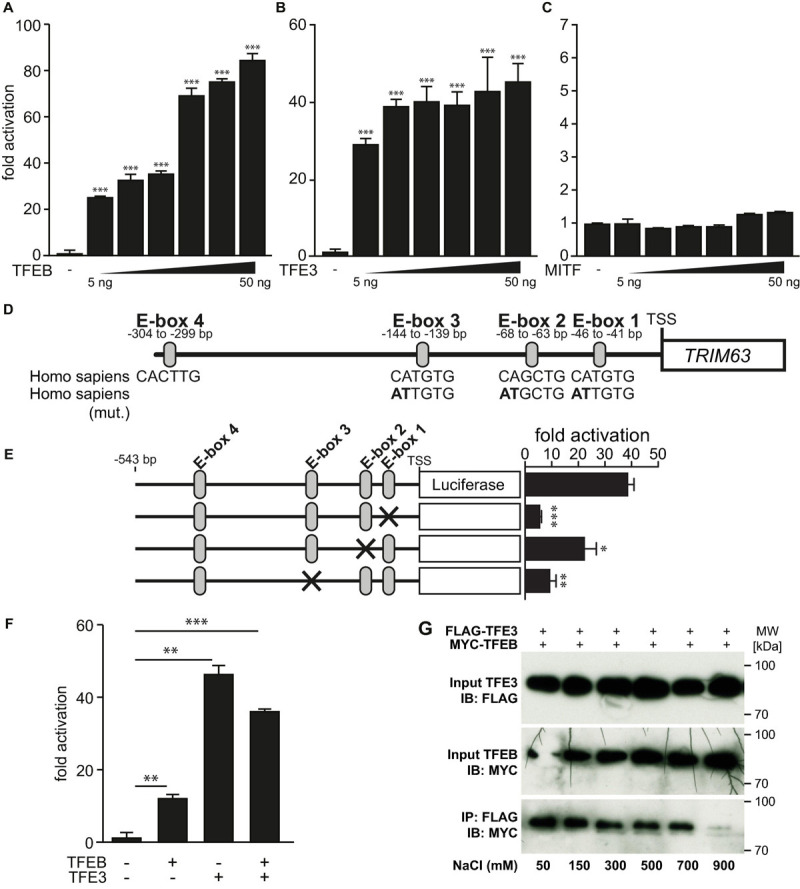
MiTF/TFE family member TFE3 but not MiTF increase *TRIM63* expression. **(A–C)** Luciferase assays performed on cell extracts of COS-7 cells transfected with Hs_*TRIM63*-Luc (-543 bp) with increasing amounts of **(A)** FLAG-TFEB (TFEB), **(B)** FLAG-TFE3, or **(C)** FLAG-MiTF as indicated or control (-) plasmid. Luciferase activity was normalized to expression of CMV-LacZ and expressed as fold increase. Data are represented as mean ± SEM. ***P < 0.005 vs. control transfection with pcDNA3.1. **(D)** Schematic diagram of the human *TRIM63*-promoter. Positions of conserved E-box motifs (CANNTG) relative to the transcription start site (TSS) are indicated. *Homo sapiens* (mut.) indicates mutated nucleotides to inactivate individual E-boxes (mutated nucleotides are shown in bold). One-way ANOVA *P* < 0.0001 **(A,B)** and n.s. **(C)**, respectively; *P < 0.05; **P < 0.01; ***P < 0.005. **(E)** COS-7 cells were transfected with a TFE3 expression plasmid and the indicated *TRIM63*-promoter constructs (–543 bp) harboring E-box mutations, as shown in **(D)**. Data are represented as mean ± SEM. *P < 0.05; **P < 0.01; ***P < 0.005. **(F)** Luciferase assays (Hs_*TRIM63*-Luc; -543 bp) performed on cell extracts of COS-7 cells after a single transfection with either MYC-TFEB (TFEB) or FLAG-TFE3 (TFE3) or cotransfection with MYC-TFEB (TFEB) and FLAG-TFE3 (TFE3). Luciferase activity was normalized to expression of CMV-LacZ and expressed as fold increase. Data are represented as mean ± SEM. *P < 0.05; **P < 0.01; ***P < 0.005. **(G)**, Co-immunoprecipitation (Co-IP) assay with lysates from COS-7 cells expressing FLAG-TFE3 and MYC-TFEB. TFE3 fusion proteins were immunoprecipitated (IP) with anti-FLAG M2 agarose, and TFEB fusion proteins were detected with an antibody directed against MYC. Input proteins were detected by Western blot (immunoblot, IB) with antibodies directed against the FLAG- or MYC-tag. *n* = 3. During cell lysis and IP increasing concentrations of sodium chloride (50, 100, 300, 500, 700, and 900 mM) were used as indicated.

Because TFEB and the closely related MiTF-family members TFE3 and MiTF elicit some degree of functional redundancy and bind to the same E-box elements (Steingrimsson et al., 2004; Martina et al., 2014; Pastore et al., 2016), we hypothesized that TFE3 and MiTF could regulate *TRIM63* expression as well. Indeed, TFEB (Figure 4A) and TFE3 (Figure 4B) but not MiTF (Figure 4C) increased *TRIM63* activity in a dose-dependent manner. Thus, for further experiments, we focused on TFE3 and excluded MiTF. Using the wild-type and E-box mutated *TRIM63* reporter constructs (Figure 4D; Du Bois et al., 2015), we found that the TFE3-induced *TRIM63* expression was strongly reduced in E-box 1 and E-box 3 mutants, whereas mutation of E-box 2 had a weaker effect (Figure 4E). These data indicate that E-box 1 and E-box 3 mediate the TFEB- and TFE3-induced *TRIM63* activity.

### Transcription Factor EB and Transcription Factor Binding to Immunoglobulin Heavy-Chain Enhancer 3 Strongly Interact With Each Other

Because TFEB and TFE3 used the same E-box motives, we investigated if TFEB and TFE3 have additive functions on the *TRIM63* promoter. We found that equimolar concentrations of TFEB and TFE3 activated the *TRIM63* promoter to comparable degrees and that their combination did not result in a further increase in *TRIM63* activity (Figure 4F). These data indicate that TFEB and TFE3 are possibly competing for the same E-box elements. As TFEB and TFE3 are known to heterodimerize, we tested the strength of this interaction. We performed cell lysis and co-immunoprecipitation with increasing sodium chloride buffer concentrations ranging from 50- to 900-mM sodium chloride. Only the highest sodium chloride concentration used attenuated the interaction between TFEB and TFE3, indicative of a strong interaction of both proteins (Figure 4G).

### Class IIa Histone Deacetylases 4, 5, and 7 Inhibit Transcription Factor Binding to Immunoglobulin Heavy-Chain Enhancer 3-Mediated *Tripartite Motif-Containing 63* Expression

**FIGURE 5:**
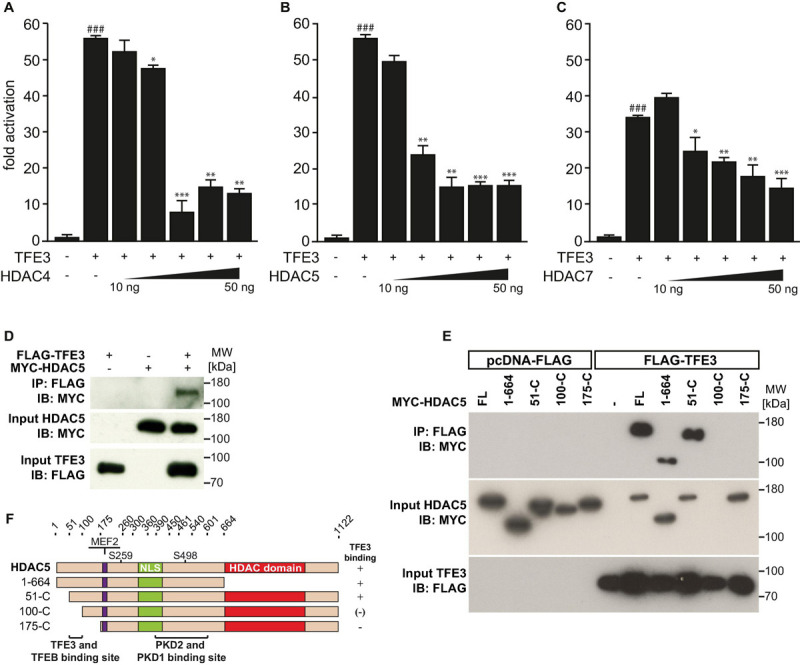
Class IIa histone deacetylases 4, 5, and 7 inhibit TFE3-mediated *TRIM63* expression. **(A–C)**, Luciferase assays performed on cell extracts of COS-7 cells transfected with Hs_*TRIM63*-Luc (-543 bp), FLAG-TFE3 (TFE3), and increasing amounts of **(A)** MYC-HDAC4 (HDAC4), **(B)** MYC-HDAC5 (HDAC5), or **(C)** MYC-HDAC7 (HDAC7) as indicated or control (-) plasmid. Luciferase activity was normalized to expression of CMV-LacZ and expressed as fold increase. Data are represented as mean ± SEM. One-way ANOVA *P* < 0.0001 for **(A–C)**; ^###^*P* < 0.005 vs. control transfection with pcDNA3.1; *P < 0.05, **P < 0.01, ***P < 0.005 vs. transfection with TFE3 only. **(D)** Co-IP assay with lysates from COS-7 cells expressing FLAG-TFE3 and MYC-HDAC5. TFE3 fusion proteins were immunoprecipitated (IP) with anti-FLAG M2 agarose, and HDAC5 fusion proteins were detected with an antibody directed against MYC. Input proteins were detected by Western blot (immunoblot, IB) with antibodies directed against the FLAG- or MYC-tag. *n* = 3. **(E)** Co-IP assay with lysates from COS-7 cells expressing FLAG-TFE3 and deletion mutants of MYC-HDAC5, as indicated. TFE3 fusion proteins were immunoprecipitated (IP) with anti-FLAG M2 agarose, and HDAC5 fusions proteins were detected with an antibody directed against MYC. Input proteins were detected by Western blot (immunoblot, IB) with antibodies directed against the FLAG- or MYC-tag. *n* = 3. **(F)**, Scheme of TFE3 binding site in HDAC5.

We next hypothesized that the activity of TFE3 on the *TRIM63* promoter is also inhibited by class IIa HDACs. Using luciferase assays, we found that HDAC4 (Figure 5A), HDAC5 (Figure 5B), and HDAC7 (Figure 5C) inhibited TFE3-induced *TRIM63* activity in a dose-dependent manner, indicating that not only TFEB but also TFE3 is collectively controlled by class IIa HDACs. In contrast, TFE3-induced *TRIM63* activity was not reduced by HDAC1 or HDAC3 (Supplementary Figure 1B). We performed co-immunoprecipitation experiments and found that TFE3 and HDAC5 interacted with each other (Figure 5D). We next performed co-immunoprecipitation experiments with TFE3 and wild-type and deletion mutants of HDAC5 to determine which region within HDAC5 was required for its interaction with TFE3. We found that amino acids 51–175 of HDAC5 are required for physical interaction with TFE3 and, therefore, define a TFE3 binding site (Figures 5E,F). We performed immunocytochemistry using antibodies directed against endogenous TFE3 and endogenous HDAC4, HDAC5, and HDAC7 and found that these proteins colocalized in C2C12 myocytes indicating that TFE3 interacts with class IIa HDACs in myocytes (Supplementary Figure 4).

### Protein Kinase D Family Members Attenuate Histone Deacetylase-Mediated Inhibition of Transcription Factor Binding to Immunoglobulin Heavy-Chain Enhancer 3-Induced *Tripartite Motif-Containing 63* Activity

**FIGURE 6:**
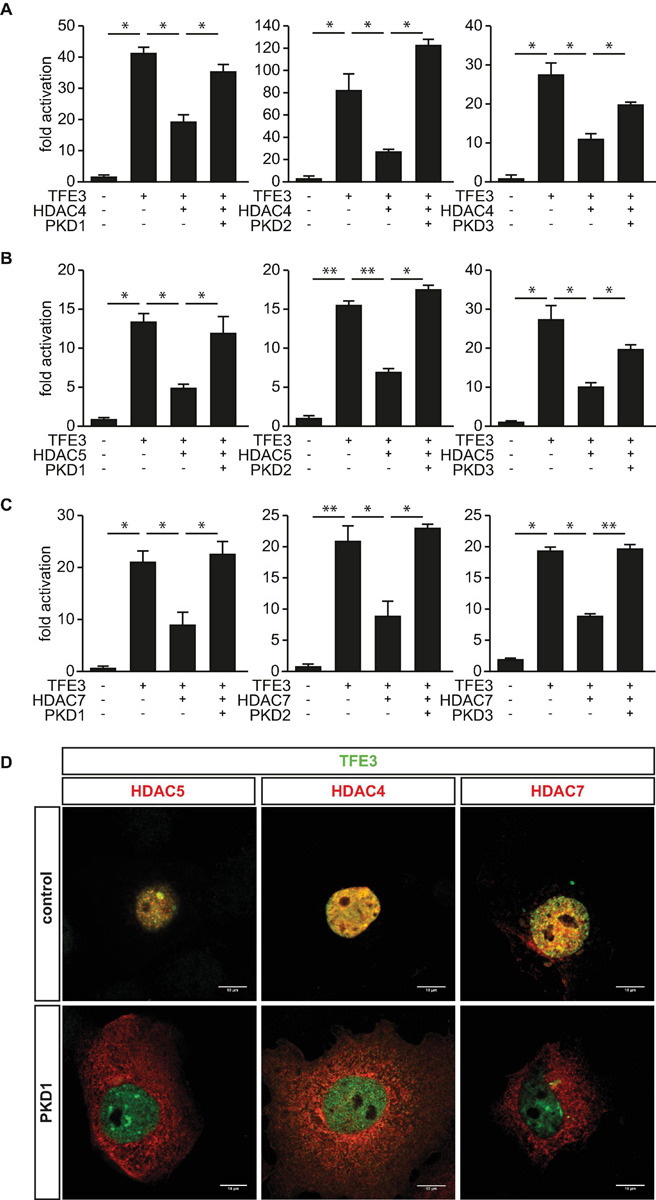
Protein kinase D family members attenuate HDAC-mediated inhibition of TFE3-induced *TRIM63* expression. **(A–C)**, COS-7 cells were transfected with expression plasmids encoding FLAG-TFE3, **(A)** HDAC4-MYC, **(B)** HDAC5-MYC, or **(C)** HDAC7-MYC, or constitutively active (ca) PKD1 (left panel), caPKD2 (middle panel), and caPKD3 (right panel) proteins, as indicated, together with the Hs_*TRIM63*_Luc reporter construct (-543 bp). Values were normalized to expression of CMV-LacZ and calculated as the fold increase in luciferase/CMV-LacZ ratio compared with the reporter alone. Data are represented as mean ± SEM. One-way ANOVA *P* < 0.0001 for **(A–C)**; *P < 0.05; **P < 0.005. *n* = 5. **(D)** COS-7 cells were transfected with FLAG-TFE3 and HDAC4-MYC, HDAC5-MYC, and HDAC7-MYC together with an empty vector (pcDNA_3.1) or caPKD1, as indicated. Immunostaining was performed with anti-FLAG (green) and anti-MYC (red) antibodies. Scale bars, 10 μm.

Based on our findings, we hypothesized that the PKD-family members could abolish the inhibitory effects of HDAC4, HDAC5, and HDAC7 toward TFE3. To test this hypothesis, we performed luciferase assays and found that the TFE3-induced *TRIM63* activity was inhibited by HDAC4 (Figure 6A), HDAC5 (Figure 6B), and HDAC7 (Figure 6C). Importantly, cotransfection of PKD1 (Figure 6, left panel), PKD2 (Figure 6, middle panel), or PKD3 (Figure 6, right panel) relieved the repressive effects of all three HDACs. CamK IV also relieved the repressive effects of HDAC4, HDAC5, and HDAC7 in the same assay (Supplementary Figure 2B). To investigate if the observed effects of the PKD family onto HDAC-mediated inhibition of TFE3 are due to nuclear export of HDAC4, HDAC5, or HDAC7, we performed immunocytochemistry experiments. We observed colocalization of all class IIa HDACs with TFE3 in the nucleus (Figure 6D). When we cotransfected with PKD1, HDAC5 (Figure 6D, left panel), HDAC4 (Figure 6D, middle panel), and HDAC7 (Figure 6D, right panel) translocated to the cytoplasm, whereas TFE3 remained in the nucleus. These data show that PKD1, PKD2, and PKD3 attenuated the inhibitory effect of HDAC4, HDAC5, and HDAC7 onto TFE3-induced *TRIM63* expression by nuclear export of these HDACs.

## Discussion

We report that *TRIM63* expression is tightly regulated by a transcriptional network comprised of the PKD-family members PKD1, PKD2, and PKD3, the class IIa HDACs HDAC4, HDAC5, and HDAC7, as well as the MiT/TFE family members TFEB and TFE3. We show that HDAC4, HDAC5, and HDAC7 colocalize with TFEB and TFE3 in the nucleus of non-muscle cells and inhibit TFEB- and TFE3-induced *TRIM63* expression. We demonstrate that PKD1, PKD2, and PKD3 phosphorylate HDAC4, HDAC5, and HDAC7. This state-of-affairs facilitates their binding to the 14-3-3 chaperon protein and their consecutive nuclear-export relieving repression of TFEB- and TFE3-induced *TRIM63* expression. All PKD family members ameliorated HDAC-mediated inhibition of TFEB- and TFE3-mediated *TRIM63* promoter activity. We conclude that *TRIM63* expression, which is a key factor in UPS-dependent protein degradation in skeletal muscle during atrophy, is regulated at multiple levels.

Skeletal muscle atrophy is characterized by a reduction in myofiber size, mainly due to a net loss of structural proteins leading to a reduced muscle mass and function. As it occurs in many pathological conditions, such as critical illness and inflammation, this results from increased UPS-dependent protein degradation (Hershko and Ciechanover, 1998; Ciechanover, 2006). However, muscle atrophy is a complex process that is mediated by multiple factors. Indeed, the molecules, mediators, and cellular pathways contributing to muscle atrophy are still being discovered (Bodine and Baehr, 2014). In 2001, Bodine et al. (2001a) identified *TRIM63*/MuRF1 as an atrogene that was highly upregulated in atrophying muscle after denervation, immobilization, and unweighting-induced atrophy in rats. MuRF1/*Trim63* knockout mice showed less denervation-induced muscle atrophy compared with wild-type mice (Bodine et al., 2001a). Thereafter, we and others confirmed the involvement of MuRF1 in muscle atrophy (Du Bois et al., 2015), described its increased expression in physiological and pathological muscle atrophy (Schmidt et al., 2014; Wollersheim et al., 2014; Huang et al., 2017), and discovered MuRF1 targets (Fielitz et al., 2007; Witt et al., 2008; Labeit et al., 2010; Nowak et al., 2019).

Increased *TRIM63* expression is a well-established and accepted read-out for skeletal muscle atrophy in general. The expression of MuRF1/*TRIM63* is regulated by several signaling pathways converging onto multiple TFs, such as the forkhead box protein family (Stitt et al., 2004; Raffaello et al., 2010) and myogenin (Moresi et al., 2010). Glass and co-workers demonstrated that the insulin-like growth factor/phosphoinositide 3-kinase/protein kinase B/Akt pathway, which had previously been shown to induce hypertrophy (Bodine et al., 2001b), suppresses atrophy by downregulating MuRF1. Myogenin was also shown to regulate *TRIM63*, and mice that lack myogenin failed to upregulate *TRIM63* expression. These mice were resistant to denervation-induced muscle atrophy (Moresi et al., 2010). Given the importance of transcriptional regulation of *TRIM63*, we previously performed a cDNA expression screen to identify regulators of *TRIM63* expression. We identified TFEB as an important TF and described its regulation and importance in muscle atrophy in myocytes *in vitro* and in muscle *in vivo* (Du Bois et al., 2015).

TFEB belongs to the MiT/TFE family of bHLH-LZ TFs, including TFE3, MiTF, and TFEC (Steingrimsson et al., 2004). All MiT/TFE TFs recognize a unique E-box motif within the proximal promoters of lysosomal and autophagy genes (Sardiello et al., 2009; Palmieri et al., 2011; Martina et al., 2014) and regulate cellular catabolism and nutrient-dependent lysosomal response (Settembre et al., 2011; Slade and Pulinilkunnil, 2017). Within the MiT/TFE family, a close relationship exists between TFEB and TFE3. TFEB and TFE3 regulate cellular adaptation to stress by induction of lysosomal biogenesis and autophagy and immune responses, metabolism, mitochondrial homeostasis, and unfolded-protein responses. TFEB and TFE3 were shown to regulate the same genes (Pastore et al., 2016), such as *CDH1*, encoding E-cadherin (Huan et al., 2005), *CD40L*, encoding CD40 ligand (Huan et al., 2006), and *PPARGC1a*, encoding PGC1α (Settembre et al., 2013). However, whether or not TFE3 activates *TRIM63* expression was not known. We report that not only TFEB but also TFE3 regulates *TRIM63* expression and that both TFs are controlled by the PKD and HDAC enzyme families. To the well-described TFEB and TFE3 functions, we add that both TFs also regulate *TRIM63* expression. We propose that they activate UPS- and autophagy-mediated protein degradation. We showed earlier that TFEB regulates *TRIM63* expression via binding to E-box elements in the *TRIM63* promoter, and mutation of these binding motifs abrogated TFEB-dependent *TRIM63* expression. However, although MiT/TFE family members bind specific E-box elements and use the same E-box motifs as TFEB to induce *TRIM63* expression, we have not yet demonstrated direct binding of TFE3 to the native *TRIM63* promoter. Such analyses need to be performed.

MuRF1 and the regulation of its expression are important for metabolic adaptations of striated muscles (Hirner et al., 2008; Witt et al., 2008; Rudolf et al., 2013). MuRF1 mediates the degradation of numerous metabolic enzymes, such as pyruvate dehydrogenase and its regulator pyruvate dehydrogenase kinase that are key mitochondrial enzymes regulating glycolysis. Interaction studies also demonstrated that MuRF1 interacts with pyruvate kinase 2, phosphorylase beta, and glycogenin, which are involved in glycolysis and glycogen metabolism, respectively (Hirner et al., 2008). Because TFEB (Mansueto et al., 2017), TFE3 (Iwasaki et al., 2012), and forkhead box (Schiaffino et al., 2013) are also involved in muscle metabolism and mitochondrial homeostasis, these effects are possibly mediated by their ability to regulate *TRIM63* expression. As our conclusions are mainly based on data obtained from non-muscle cells, this hypothesis warrants further investigation to show its relevance in muscle metabolism.

Distinguished by their structures and expression patterns, HDACs can be divided into different classes. We focused on the heart and skeletal muscle enriched class IIa HDACs 4, 5, and 7. All class IIa HDACs repress the activity of myocyte enhancer factor 2 (MEF2), which reduces the expression of MEF2 target genes and suppresses the formation of slow-twitch, oxidative fibers in the muscle (Lu et al., 2000a,b; McKinsey et al., 2000; Zhang et al., 2002; Potthoff et al., 2007). HDAC4 and HDAC5 coordinately inhibited the activity of myogenin that is important for myogenesis (Moresi et al., 2010). Latter data implicated that HDAC4, HDAC5, and HDAC7 predominantly inhibit bHLH TFs contained in muscle to regulate muscular stress response. However, if other class IIa HDACs inhibit TFEB and if they can also reduce TFE3-mediated *TRIM63* expression was unknown. We describe that HDAC4, HDAC5, and HDAC7 inhibit TFEB-mediated *TRIM63* activity and show that all three HDACs inhibited TFE3. We propose that class IIa HDACs collectively control *TRIM63* expression. However, as our experiments were mainly performed in non-myocytes, further studies are needed to investigate if this mode of action also occurs in myocytes.

Protein function is often regulated by reversible protein phosphorylation by protein kinases, such as the PKD family (Rykx et al., 2003; Rozengurt, 2011). This family of stress-responsive serine-threonine kinases of the calmodulin-calcium-dependent-kinase family (termed PKD1, PKD2, and PKD3) play important roles in cell growth, differentiation, migration, and apoptosis (Rozengurt et al., 2005). Importantly, we and others showed that PKD1 mediates muscle atrophy (Du Bois et al., 2015), facilitates slow-twitch-fiber transformation in the muscle (Kim et al., 2008), and mediates cardiac stress response (Fielitz et al., 2008). However, the close structural relationship of the PKD family members especially in their functional domains (Avkiran et al., 2008) suggested that they might phosphorylate overlapping targets and might be involved in the observed phenotypes as well. Indeed, PKD1, PKD2, and PKD3 were all shown to phosphorylate HDAC5 (Huynh and McKinsey, 2006). This situation, in turn, relieves the repression of TFs such as MEF2 and TFEB (Fielitz et al., 2008; Du Bois et al., 2015). The mechanism is involved in the pathophysiology of cardiomyocyte hypertrophy *in vitro* and cardiac hypertrophy and remodeling *in vivo* (Vega et al., 2004; Fielitz et al., 2008). These observations suggested that PKD family members redundantly control the activity of class IIa HDACs (Huynh and McKinsey, 2006). However, it was uncertain if this interaction affects TFEB- or TFE3-mediated *TRIM63* expression. We confirmed this hypothesis for all three PKD family members and show that they phosphorylate HDAC4, HDAC5, and HDAC7 and promote their nuclear export. Because a high degree of functional redundancy was reported for PKD1 and PKD2, we investigated if PKD2 also interacts with HDAC5. We confirmed this interaction and mapped the binding of PKD2 to HDAC5 to the same region in HDAC5 that binds to PKD1, suggesting that *TRIM63* expression is redundantly controlled. We also show that PKD3, which is normally not expressed in unstressed myocytes (Li et al., 2011), elicits comparable effects toward the control of *TRIM63* expression by class IIa HDACs. This state-of-affair might especially be important during stress situations where PKD3 was shown to be strongly increased (Li et al., 2011). Our data are in line with previous reports showing that PKD3 can substitute for PKD1 as an HDAC5 kinase in non-muscle cells (Matthews et al., 2006). In summary, our data indicate that a diverse and multilevel pathway regulates muscle atrophy.

Subcellular localization of class IIa HDACs is strongly controlled by several protein kinases, which are not restricted to the protein kinase D family but also involve CamK I, CamK IV (also shown here), MARK2, and others (Chang et al., 2005). Also, some kinases selectively target specific class IIa HDACs, i.e., CamK II specifically targets HDAC4 (Backs et al., 2006). These data, together with our findings and the known regulation of TFEB and TFE3 activity by posttranslational modification (Puertollano et al., 2018), indicate that TFEB- and TFE3-mediated transcriptional activity has a much higher level of complexity that could not be addressed here and warrants further studies.

Previous studies showed that TFEB (Settembre et al., 2012) and TFE3 (Martina et al., 2014) are predominantly localized in the cytoplasm of HEK-293T and ARPE-19 cells, respectively, and that their phosphorylation status and cytosolic-to-nuclear shuttling regulates TFEB and TFE3 activity. The kinases mammalian target of rapamycin complex 1 (mTORC1) and ERK are important in that regard (Martina et al., 2012, 2014; Settembre et al., 2012). In the presence of nutrients, mTORC1 phosphorylates TFEB and TFE3, thereby facilitating their binding to 14-3-3 chaperone proteins and mediating their retention in the cytoplasm. Conversely, reduced mTORC1 activity increases TFEB (Settembre et al., 2012) and TFE3 (Martina et al., 2014) shuttling into the nucleus. In contrast, in line with our previous report (Du Bois et al., 2015), we observed that TFEB and TFE3 were mainly localized to the nucleus of COS-7 cells when overexpressed. If these observations are attributable to differences in the cell type, culturing conditions, differentiation status, or transfection warrants further investigation. However, our observation that TFEB and TFE3 are localized to the nucleus is supported by other findings showing that 20–30% of TFEB is contained in the nucleus of several cell lines, such as patient-derived fibroblasts (Song et al., 2013), HeLa cells (Settembre et al., 2011), ARPE-19 cells (Martina et al., 2012), and mouse embryonic fibroblasts (Sardiello et al., 2009). Because we hypothesized that HDAC4, HDAC5, and HDAC7 regulate the activity of TFEB and TFE3 at the *TRIM63* promoter, as reported for their inhibitory effects on MEF2 target genes (Lu et al., 2000a,b; McKinsey et al., 2000; Zhang et al., 2002), we used overexpression as a model system despite differences in subcellular localization of the participating proteins. Our data indicate that the activity of TFEB and TFE3 is regulated at least at two different levels, first by regulation of their subcellular localization and second by repression of their activity by class IIa HDACs.

## Limitations

Most of the data shown in our study are based on overexpression experiments. Our results need further evaluation, especially in myocytes, myotubes, and skeletal muscle, and by working with the endogenous components of the signaling pathway described, such as co-staining of endogenous proteins in immunocytochemistry. Nevertheless, our results are in line with previously published work that focused on the regulation of the activity and subcellular localization of class IIa HDACs by PKD both *in vitro* (Zhang et al., 2002; Dequiedt et al., 2005; Backs et al., 2006) and *in vivo* (Fielitz et al., 2008; Kim et al., 2008; Du Bois et al., 2015). Although we have shown that upon coexpression with PKD, HDAC4, HDAC5, and HDAC7 are localized in the cytoplasm, and PKD increased UAS-luciferase activity that depends on 14-3-3 binding, we have not proven that this export was mediated by CRM1. Likewise, to illustrate our findings, we display representative pictures of single nuclei and have not performed biochemical fractionation experiments (nuclear vs. cytosolic extracts) to support the nuclear export of the HDACs upon PKD1 overexpression for a larger subset of cells. Because such experiments have been performed previously, we would like to refer to this work (Harrison et al., 2004; Vega et al., 2004). We have used co-immunoprecipitation experiments to show the interaction of PKD1 and PKD2 with HDAC5, mapped the interacting domains, and showed functional consequences of these interactions. We have also shown that the activity of TFEB and TFE3 on the *TRIM63* reporter is inhibited by class IIa HDACs. However, further studies are needed to prove that these proteins directly interact which each other by using cell-free assays, proximity ligation assays, coimmunostaining, or other techniques.

## Data Availability Statement

The original contributions presented in the study are included in the article/Supplementary Material, further inquiries can be directed to the corresponding author/s.

## Author Contributions

CP and BF designed and performed experiments, prepared figures, and prepared the manuscript. YL performed experiments, prepared figures, and provided intellectual input. JR designed experiments and discussed data. FL discussed data, provided intellectual input, and rewrote the manuscript. JF supervised the project, analyzed data, and rewrote the manuscript. All authors contributed to the article and approved the submitted version.

## Conflict of Interest

The authors declare that the research was conducted in the absence of any commercial or financial relationships that could be construed as a potential conflict of interest.

## Abbreviations

HDAC: histone deacetylase-5
MuRF: Muscle really interesting new gene (RING)-finger containing protein
MiT/TFE: Microphthalmia-associated transcription factor/transcription factor E
PKD: Protein Kinase D
TF: transcription factor
TFEB: transcription factor EB
TFE3: Transcription factor for immunoglobulin heavy-chain enhancer 3
*TRIM63*: Tripartite motif-containing 63 (mouse gene encoding MuRF1).

## References

[B1] Avkiran M. Rowland A. J. Cuello F. Haworth R. S. (2008). Protein kinase d in the cardiovascular system: emerging roles in health and disease. *Circ. Res.* 102 157–163. 10.1161/circresaha.107.168211 18239146

[B2] Backs J. Song K. Bezprozvannaya S. Chang S. Olson E. N. (2006). CaM kinase II selectively signals to histone deacetylase 4 during cardiomyocyte hypertrophy. *J. Clin. Invest.* 116 1853–1864. 10.1172/jci27438 16767219 PMC1474817

[B3] Bodine S. C. Baehr L. M. (2014). Skeletal muscle atrophy and the E3 ubiquitin ligases MuRF1 and MAFbx/atrogin-1. *Am. J. Physiol. Endocrinol. Metab.* 307 E469–E484. 25096180 10.1152/ajpendo.00204.2014 PMC4166716

[B4] Bodine S. C. Latres E. Baumhueter S. Lai V. K. Nunez L. Clarke B. A. (2001a). Identification of ubiquitin ligases required for skeletal muscle atrophy. *Science* 294 1704–1708. 10.1126/science.1065874 11679633

[B5] Bodine S. C. Stitt T. N. Gonzalez M. Kline W. O. Stover G. L. Bauerlein R. (2001b). Akt/mTOR pathway is a crucial regulator of skeletal muscle hypertrophy and can prevent muscle atrophy in vivo. *Nat. Cell Biol.* 3 1014–1019. 10.1038/ncb1101-1014 11715023

[B6] Chang S. Bezprozvannaya S. Li S. Olson E. N. (2005). An expression screen reveals modulators of class II histone deacetylase phosphorylation. *Proc. Natl. Acad. Sci. U.S.A.* 102 8120–8125. 10.1073/pnas.0503275102 15923258 PMC1149448

[B7] Ciechanover A. (2006). The ubiquitin proteolytic system: from a vague idea, through basic mechanisms, and onto human diseases and drug targeting. *Neurology* 66 S7–S19. 16432150 10.1212/01.wnl.0000192261.02023.b8

[B8] Dequiedt F. Van Lint J. Lecomte E. Van Duppen V. Seufferlein T. Vandenheede J. R. (2005). Phosphorylation of histone deacetylase 7 by protein kinase D mediates T cell receptor-induced Nur77 expression and apoptosis. *J. Exp. Med.* 201 793–804. 10.1084/jem.20042034 15738054 PMC2212830

[B9] Du Bois P. Pablo Tortola C. Lodka D. Kny M. Schmidt F. Song K. (2015). Angiotensin II Induces Skeletal Muscle Atrophy by Activating TFEB-Mediated MuRF1 Expression. *Circ. Res.* 117 424–436. 10.1161/circresaha.114.305393 26137861 PMC4537692

[B10] Emiliani S. Fischle W. Van Lint C. Al-Abed Y. Verdin E. (1998). Characterization of a human RPD3 ortholog, HDAC3. *Proc. Natl. Acad. Sci. U.S.A.* 95 2795–2800. 10.1073/pnas.95.6.2795 9501169 PMC19648

[B11] Fielitz J. Kim M. S. Shelton J. M. Latif S. Spencer J. A. Glass D. J. (2007). Myosin accumulation and striated muscle myopathy result from the loss of muscle RING finger 1 and 3. *J. Clin. Invest.* 117 2486–2495. 10.1172/jci32827 17786241 PMC1957544

[B12] Fielitz J. Kim M. S. Shelton J. M. Qi X. Hill J. A. Richardson J. A. (2008). Requirement of protein kinase D1 for pathological cardiac remodeling. *Proc. Natl. Acad. Sci. U.S.A.* 105 3059–3063. 10.1073/pnas.0712265105 18287012 PMC2268584

[B13] Harrison B. C. Roberts C. R. Hood D. B. Sweeney M. Gould J. M. Bush E. W. (2004). The CRM1 nuclear export receptor controls pathological cardiac gene expression. *Mol. Cell. Biol.* 24 10636–10649. 10.1128/mcb.24.24.10636-10649.2004 15572669 PMC533968

[B14] Hershko A. Ciechanover A. (1998). The ubiquitin system. *Annu. Rev. Biochem.* 67 425–479. 9759494 10.1146/annurev.biochem.67.1.425

[B15] Hirner S. Krohne C. Schuster A. Hoffmann S. Witt S. Erber R. (2008). MuRF1-dependent regulation of systemic carbohydrate metabolism as revealed from transgenic mouse studies. *J. Mol. Biol.* 379 666–677. 10.1016/j.jmb.2008.03.049 18468620

[B16] Huan C. Kelly M. L. Steele R. Shapira I. Gottesman S. R. Roman C. A. (2006). Transcription factors TFE3 and TFEB are critical for CD40 ligand expression and thymus-dependent humoral immunity. *Nat. Immunol.* 7 1082–1091. 10.1038/ni1378 16936731 PMC2386253

[B17] Huan C. Sashital D. Hailemariam T. Kelly M. L. Roman C. A. (2005). Renal carcinoma-associated transcription factors TFE3 and TFEB are leukemia inhibitory factor-responsive transcription activators of E-cadherin. *J. Biol. Chem.* 280 30225–30235. 10.1074/jbc.m502380200 15994295

[B18] Huang N. Kny M. Riediger F. Busch K. Schmidt S. Luft F. C. (2017). Deletion of Nlrp3 protects from inflammation-induced skeletal muscle atrophy. *Intensive Care Med. Exp.* 5:3. 10.1186/2044-5040-1-3 28097512 PMC5241267

[B19] Huynh Q. K. McKinsey T. A. (2006). Protein kinase D directly phosphorylates histone deacetylase 5 via a random sequential kinetic mechanism. *Arch. Biochem. Biophys.* 450 141–148. 10.1016/j.abb.2006.02.014 16584705

[B20] Iwasaki H. Naka A. Iida K. T. Nakagawa Y. Matsuzaka T. Ishii K. A. (2012). TFE3 regulates muscle metabolic gene expression, increases glycogen stores, and enhances insulin sensitivity in mice. *Am. J. Physiol. Endocrinol. Metab.* 302 E896–E902. 22297304 10.1152/ajpendo.00204.2011

[B21] Kedar V. Mcdonough H. Arya R. Li H. H. Rockman H. A. Patterson C. (2004). Muscle-specific RING finger 1 is a bona fide ubiquitin ligase that degrades cardiac troponin I. *Proc. Natl. Acad. Sci. U.S.A.* 101 18135–18140. 10.1073/pnas.0404341102 15601779 PMC539735

[B22] Kim M. S. Fielitz J. Mcanally J. Shelton J. M. Lemon D. D. Mckinsey T. A. (2008). Protein kinase D1 stimulates MEF2 activity in skeletal muscle and enhances muscle performance. *Mol. Cell. Biol.* 28 3600–3609. 10.1128/mcb.00189-08 18378694 PMC2423294

[B23] Koyama S. Hata S. Witt C. C. Ono Y. Lerche S. Ojima K. (2008). Muscle RING-finger protein-1 (MuRF1) as a connector of muscle energy metabolism and protein synthesis. *J. Mol. Biol.* 376 1224–1236. 10.1016/j.jmb.2007.11.049 18222470

[B24] Labeit S. Kohl C. H. Witt C. C. Labeit D. Jung J. Granzier H. (2010). Modulation of muscle atrophy, fatigue and MLC phosphorylation by MuRF1 as indicated by hindlimb suspension studies on MuRF1-KO mice. *J. Biomed. Biotechnol.* 2010:693741. 10.1155/2010/693741 PMC2896721 20625437

[B25] Langhans C. Weber-Carstens S. Schmidt F. Hamati J. Kny M. Zhu X. (2014). Inflammation-induced acute phase response in skeletal muscle and critical illness myopathy. *PLoS One* 9:e92048. 10.1371/journal.pone.0092048 24651840 PMC3961297

[B26] Li C. Li J. Cai X. Sun H. Jiao J. Bai T. (2011). Protein kinase D3 is a pivotal activator of pathological cardiac hypertrophy by selectively increasing the expression of hypertrophic transcription factors. *J. Biol. Chem.* 286 40782–40791. 10.1074/jbc.m111.263046 21971046 PMC3220477

[B27] Lodka D. Pahuja A. Geers-Knorr C. Scheibe R. J. Nowak M. Hamati J. (2016). Muscle RING-finger 2 and 3 maintain striated-muscle structure and function. *J. Cachexia Sarcopenia Muscle* 7 165–180. 10.1002/jcsm.12057 27493870 PMC4863828

[B28] Lu J. Mckinsey T. A. Nicol R. L. Olson E. N. (2000a). Signal-dependent activation of the MEF2 transcription factor by dissociation from histone deacetylases. *Proc. Natl. Acad. Sci. U.S.A.* 97 4070–4075. 10.1073/pnas.080064097 10737771 PMC18151

[B29] Lu J. Mckinsey T. A. Zhang C. L. Olson E. N. (2000b). Regulation of skeletal myogenesis by association of the MEF2 transcription factor with class II histone deacetylases. *Mol. Cell.* 6 233–244. 10.1016/s1097-2765(00)00025-3 10983972

[B30] Mansueto G. Armani A. Viscomi C. D’orsi L. De Cegli R. Polishchuk E. V. (2017). Transcription Factor EB Controls Metabolic Flexibility during Exercise. *Cell Metab* 25 182–196. 10.1016/j.cmet.2016.11.003 28011087 PMC5241227

[B31] Martina J. A. Chen Y. Gucek M. Puertollano R. (2012). MTORC1 functions as a transcriptional regulator of autophagy by preventing nuclear transport of TFEB. *Autophagy* 8 903–914. 10.4161/auto.19653 22576015 PMC3427256

[B32] Martina J. A. Diab H. I. Lishu L. Jeong A. L. Patange S. Raben N. (2014). The nutrient-responsive transcription factor TFE3 promotes autophagy, lysosomal biogenesis, and clearance of cellular debris. *Sci. Signal.* 7:ra9. 10.1126/scisignal.2004754 24448649 PMC4696865

[B33] Matthews S. A. Liu P. Spitaler M. Olson E. N. Mckinsey T. A. Cantrell D. A. (2006). Essential role for protein kinase D family kinases in the regulation of class II histone deacetylases in B lymphocytes. *Mol. Cell. Biol.* 26 1569–1577. 10.1128/mcb.26.4.1569-1577.2006 16449666 PMC1367196

[B34] McElhinny A. S. Kakinuma K. Sorimachi H. Labeit S. Gregorio C. C. (2002). Muscle-specific RING finger-1 interacts with titin to regulate sarcomeric M-line and thick filament structure and may have nuclear functions via its interaction with glucocorticoid modulatory element binding protein-1. *J. Cell Biol.* 157 125–136. 10.1083/jcb.200108089 11927605 PMC2173255

[B35] McKinsey T. A. Zhang C. L. Lu J. Olson E. N. (2000). Signal-dependent nuclear export of a histone deacetylase regulates muscle differentiation. *Nature* 408 106–111. 10.1038/35040593 11081517 PMC4459600

[B36] Medina R. Wing S. S. Goldberg A. L. (1995). Increase in levels of polyubiquitin and proteasome mRNA in skeletal muscle during starvation and denervation atrophy. *Biochem. J.* 307(Pt 3), 631–637. 10.1042/bj3070631 7741690 PMC1136697

[B37] Moresi V. Williams A. H. Meadows E. Flynn J. M. Potthoff M. J. Mcanally J. (2010). Myogenin and class II HDACs control neurogenic muscle atrophy by inducing E3 ubiquitin ligases. *Cell* 143 35–45. 10.1016/j.cell.2010.09.004 20887891 PMC2982779

[B38] Nowak M. Suenkel B. Porras P. Migotti R. Schmidt F. Kny M. (2019). DCAF8, a novel MuRF1 interaction partner, promotes muscle atrophy. *J. Cell Sci.* 132:JCS233395. 10.1242/jcs.233395 31391242

[B39] Palmieri M. Impey S. Kang H. Di Ronza A. Pelz C. Sardiello M. (2011). Characterization of the CLEAR network reveals an integrated control of cellular clearance pathways. *Hum. Mol. Genet.* 20 3852–3866. 10.1093/hmg/ddr306 21752829

[B40] Pastore N. Brady O. A. Diab H. I. Martina J. A. Sun L. Huynh T. (2016). TFEB and TFE3 cooperate in the regulation of the innate immune response in activated macrophages. *Autophagy* 12 1240–1258. 10.1080/15548627.2016.1179405 27171064 PMC4968228

[B41] Polge C. Cabantous S. Deval C. Claustre A. Hauvette A. Bouchenot C. (2018). A muscle-specific MuRF1-E2 network requires stabilization of MuRF1-E2 complexes by telethonin, a newly identified substrate. *J. Cachexia Sarcopenia Muscle* 9 129–145. 10.1002/jcsm.12249 29271608 PMC5803617

[B42] Polge C. Heng A. E. Jarzaguet M. Ventadour S. Claustre A. Combaret L. (2011). Muscle actin is polyubiquitinylated in vitro and in vivo and targeted for breakdown by the E3 ligase MuRF1. *FASEB J.* 25 3790–3802. 10.1096/fj.11-180968 21764995

[B43] Potthoff M. J. Wu H. Arnold M. A. Shelton J. M. Backs J. Mcanally J. (2007). Histone deacetylase degradation and MEF2 activation promote the formation of slow-twitch myofibers. *J. Clin. Invest.* 117 2459–2467. 10.1172/jci31960 17786239 PMC1957540

[B44] Puertollano R. Ferguson S. M. Brugarolas J. Ballabio A. (2018). The complex relationship between TFEB transcription factor phosphorylation and subcellular localization. *EMBO J.* 37:e98804. 10.15252/embj.201798804 PMC5983138 29764979

[B45] Raffaello A. Milan G. Masiero E. Carnio S. Lee D. Lanfranchi G. (2010). JunB transcription factor maintains skeletal muscle mass and promotes hypertrophy. *J. Cell Biol.* 191 101–113. 10.1083/jcb.201001136 20921137 PMC2953439

[B46] Rozengurt E. (2011). Protein kinase D signaling: multiple biological functions in health and disease. *Physiology* 26 23–33. 10.1152/physiol.00037.2010 21357900 PMC4381749

[B47] Rozengurt E. Rey O. Waldron R. T. (2005). Protein kinase D signaling. *J. Biol. Chem.* 280 13205–13208. 15701647 10.1074/jbc.R500002200

[B48] Rudolf R. Bogomolovas J. Strack S. Choi K. R. Khan M. M. Wagner A. (2013). Regulation of nicotinic acetylcholine receptor turnover by MuRF1 connects muscle activity to endo/lysosomal and atrophy pathways. *Age* 35 1663–1674. 10.1007/s11357-012-9468-9 22956146 PMC3776120

[B49] Rykx A. De Kimpe L. Mikhalap S. Vantus T. Seufferlein T. Vandenheede J. R. (2003). Protein kinase D: a family affair. *FEBS Lett.* 546 81–86. 10.1016/s0014-5793(03)00487-3 12829240

[B50] Sardiello M. Palmieri M. Di Ronza A. Medina D. L. Valenza M. Gennarino V. A. (2009). A gene network regulating lysosomal biogenesis and function. *Science* 325 473–477. 10.1126/science.1174447 19556463

[B51] Schiaffino S. Dyar K. A. Ciciliot S. Blaauw B. Sandri M. (2013). Mechanisms regulating skeletal muscle growth and atrophy. *FEBS J.* 280 4294–4314. 10.1111/febs.12253 23517348

[B52] Schmidt F. Kny M. Zhu X. Wollersheim T. Persicke K. Langhans C. (2014). The E3 ubiquitin ligase TRIM62 and inflammation-induced skeletal muscle atrophy. *Crit. Care* 18:545. 10.1186/s13054-014-0545-6 PMC4231194 25263070

[B53] Settembre C. De Cegli R. Mansueto G. Saha P. K. Vetrini F. Visvikis O. (2013). TFEB controls cellular lipid metabolism through a starvation-induced autoregulatory loop. *Nat. Cell Biol.* 15 647–658. 10.1038/ncb2718 23604321 PMC3699877

[B54] Settembre C. Di Malta C. Polito V. A. Garcia Arencibia M. Vetrini F. Erdin S. (2011). TFEB links autophagy to lysosomal biogenesis. *Science* 332 1429–1433. 10.1126/science.1204592 21617040 PMC3638014

[B55] Settembre C. Zoncu R. Medina D. L. Vetrini F. Erdin S. Huynh T. (2012). A lysosome-to-nucleus signalling mechanism senses and regulates the lysosome via mTOR and TFEB. *EMBO J.* 31 1095–1108. 10.1038/emboj.2012.32 22343943 PMC3298007

[B56] Slade L. Pulinilkunnil T. (2017). The MiTF/TFE family of transcription factors: master regulators of organelle signaling, metabolism, and stress adaptation. *Mol. Cancer Res.* 15 1637–1643. 10.1158/1541-7786.mcr-17-0320 28851811

[B57] Song K. Backs J. Mcanally J. Qi X. Gerard R. D. Richardson J. A. (2006). The transcriptional coactivator CAMTA2 stimulates cardiac growth by opposing class II histone deacetylases. *Cell* 125 453–466. 10.1016/j.cell.2006.02.048 16678093

[B58] Song W. Wang F. Savini M. Ake A. Di Ronza A. Sardiello M. (2013). TFEB regulates lysosomal proteostasis. *Hum. Mol. Genet.* 22 1994–2009. 10.1093/hmg/ddt052 23393155

[B59] Steingrimsson E. Copeland N. G. Jenkins N. A. (2004). Melanocytes and the microphthalmia transcription factor network. *Annu. Rev. Genet.* 38 365–411. 15568981 10.1146/annurev.genet.38.072902.092717

[B60] Stitt T. N. Drujan D. Clarke B. A. Panaro F. Timofeyva Y. Kline W. O. (2004). The IGF-1/PI3K/Akt pathway prevents expression of muscle atrophy-induced ubiquitin ligases by inhibiting FOXO transcription factors. *Mol. Cell.* 14 395–403. 10.1016/s1097-2765(04)00211-4 15125842

[B61] Vega R. B. Harrison B. C. Meadows E. Roberts C. R. Papst P. J. Olson E. N. (2004). Protein kinases C and D mediate agonist-dependent cardiac hypertrophy through nuclear export of histone deacetylase 5. *Mol. Cell. Biol.* 24 8374–8385. 10.1128/mcb.24.19.8374-8385.2004 15367659 PMC516754

[B62] Voisin L. Breuille D. Combaret L. Pouyet C. Taillandier D. Aurousseau E. (1996). Muscle wasting in a rat model of long-lasting sepsis results from the activation of lysosomal, Ca2+ -activated, and ubiquitin-proteasome proteolytic pathways. *J. Clin. Invest.* 97 1610–1617. 10.1172/jci118586 8601625 PMC507224

[B63] Wing S. S. Haas A. L. Goldberg A. L. (1995). Increase in ubiquitin-protein conjugates concomitant with the increase in proteolysis in rat skeletal muscle during starvation and atrophy denervation. *Biochem. J.* 307(Pt 3), 639–645. 10.1042/bj3070639 7741691 PMC1136698

[B64] Witt C. C. Witt S. H. Lerche S. Labeit D. Back W. Labeit S. (2008). Cooperative control of striated muscle mass and metabolism by MuRF1 and MuRF2. *EMBO J.* 27 350–360. 10.1038/sj.emboj.7601952 18157088 PMC2168395

[B65] Witt S. H. Granzier H. Witt C. C. Labeit S. (2005). MURF-1 and MURF-2 target a specific subset of myofibrillar proteins redundantly: towards understanding MURF-dependent muscle ubiquitination. *J. Mol. Biol.* 350 713–722. 10.1016/j.jmb.2005.05.021 15967462

[B66] Wollersheim T. Woehlecke J. Krebs M. Hamati J. Lodka D. Luther-Schroeder A. (2014). Dynamics of myosin degradation in intensive care unit-acquired weakness during severe critical illness. *Intensive Care Med.* 40 528–538. 10.1007/s00134-014-3224-9 24531339

[B67] Xie J. Black D. L. (2001). A CaMK IV responsive RNA element mediates depolarization-induced alternative splicing of ion channels. *Nature* 410 936–939. 10.1038/35073593 11309619

[B68] Zhang C. L. Mckinsey T. A. Chang S. Antos C. L. Hill J. A. Olson E. N. (2002). Class II histone deacetylases act as signal-responsive repressors of cardiac hypertrophy. *Cell* 110 479–488. 10.1016/s0092-8674(02)00861-9 12202037 PMC4459650

[B69] Zhu X. Kny M. Schmidt F. Hahn A. Wollersheim T. Kleber C. (2017). Secreted Frizzled-Related Protein 2 and Inflammation-Induced Skeletal Muscle Atrophy. *Crit. Care Med* 45 e169–e183. 27661566 10.1097/CCM.0000000000002056

