# Supplementary material for: Activation of Tripartite Motif Containing 63 Expression by Transcription Factor EB and Transcription Factor Binding to Immunoglobulin Heavy Chain Enhancer 3 Is Regulated by Protein Kinase D and Class IIa Histone Deacetylases
Source: Front Physiol. 2021 Jan 13;11:550506. doi: 10.3389/fphys.2020.550506 (PMC7838639; doi:10.3389/fphys.2020.550506)
Supplement: Supplementary file 1 [file Data_Sheet_1.PDF]

Supplementary Figures

Supplementary Figure 1. Class I HDACs (HDAC1 and HDAC3) do not repress TFEB or TFE3 induced *TRIM63* expression. (**A**), COS-7 cells were transfected with expression plasmids encoding FLAG-TFEB, FLAG-HDAC1 (left panel) or FLAG-HDAC3 (right panel), as indicated, together with the Hs_*TRIM63*_Luc reporter construct (−543 bp). Values were normalized to expression of CMV-LacZ and calculated as the fold-increase in luciferase/CMV-LacZ ratio compared with the reporter alone. Data are represented as mean±SEM. One-way ANOVA $P<0.0001$ for all, except $P=0.0048$ (A, left panel); *$P<0.05$. n=3. (**B**), COS-7 cells were transfected with expression plasmids encoding FLAG-TFE3, FLAG-HDAC1 (left panel) or FLAG-HDAC3 (right panel), as indicated, together with the Hs_*TRIM63*_Luc reporter construct (−543 bp). Values were normalized to expression of CMV-LacZ and calculated as the fold-increase in luciferase/CMV-LacZ ratio compared with the reporter alone. Data are represented as mean±SEM. One-way ANOVA $P<0.0001$ for both; *$P<0.05$. n=3.

Supplementary Figure 2. Calcium/calmodulin-dependent protein kinase IV (CamK IV) attenuates HDAC-mediated inhibition of TFEB and TFE3-induced *TRIM63* expression. (**A**), COS-7 cells were transfected with expression plasmids encoding FLAG-TFEB, (left panel), HDAC4-MYC, (middle panel), HDAC5-MYC or (right panel), HDAC7-MYC, as indicated, together with the Hs_*TRIM63*_Luc reporter construct (−543 bp). Values were normalized to expression of CMV-LacZ and calculated as the fold-increase in luciferase/CMV-LacZ ratio compared with the reporter alone. Data are represented as mean±SEM. One-way ANOVA $P<0.0001$ for all; *$P<0.05$; **$P<0.01$. n=3. (**B**), COS-7 cells were transfected with expression plasmids encoding FLAG-TFE3, (left panel), HDAC4-MYC, (middle panel), HDAC5-MYC or (right panel), HDAC7-MYC, as indicated, together with the Hs_*TRIM63*_Luc reporter construct (−543 bp). Values were normalized to expression of CMV-

LacZ and calculated as the fold-increase in luciferase/CMV-LacZ ratio compared with the reporter alone. Data are represented as mean±SEM. One-way ANOVA $P<0.0001$ for all; *$P<0.05$; **$P<0.01$. n=3.

Supplementary Figure 3. Overexpressed PKD phosphorylate endogenous class IIa HDACs. (**A, top**), C2C12 myoblasts were transfected with PKD1, PKD2 or PKD3 cDNA expression plasmids for 24 hours. pcDNA transfected cells served as controls. Western blot analyses of isolated proteins with anti-phospho-HDAC4 (Ser246)/HDAC5 (Ser259)/HDAC7 (Ser155), anti-phospho-HDAC4 (Ser632)/HDAC5 (Ser661)/HDAC7 (Ser486), anti-HDAC4, anti-HDAC5 and anti-HDAC7 are shown. GAPDH was used as loading control. **(A, bottom)**, Bar graph showing the relative densities of protein contents of phospho-HDAC4 (Ser246)/HDAC5 (Ser259), phosphor-HDAC7 (Ser155) and phospho-HDAC4 (Ser632)/HDAC5 (Ser661) as detected in A. Signal intensity of control transfected cells was set to 1. (**B, top**), C2C12 myoblasts were transfected with PKD1 or pcDNA control for 24 hours, and afterwards treated with the PKD inhibitor CID 2011756 (50 μM) or vehicle (DMSO) for 6 hours. Western blot analyses of isolated proteins with anti-phospho-HDAC4 (Ser246)/HDAC5 (Ser259)/HDAC7 (Ser155), anti-phospho-HDAC4 (Ser632)/HDAC5 (Ser661)/HDAC7 (Ser486), anti-HDAC4, anti-HDAC5 and anti-HDAC7 are shown. GAPDH was used as loading control. **(B, bottom)**, Bar graph showing the relative densities of protein contents of phospho-HDAC4 (Ser246)/HDAC5 (Ser259), phospho-HDAC7 (Ser155), phospho-HDAC4 (Ser632)/HDAC5 (Ser661) and phospho-HDAC7 (Ser486) as detected in A. Signal intensity of control transfected cells was set to 1.

Supplementary Figure 4. Endogenous TFE3 and class IIa HDACs colocalize in C2C12 myoblasts. Immunohistochemistry of C2C12 myoblasts with anti-TFE3 (green at 488 nm) and

anti-HDAC4, anti-HDAC5 or anti-HDAC7 antibody (all red at 555 nm), as indicated. The scale bar, 50 μm.

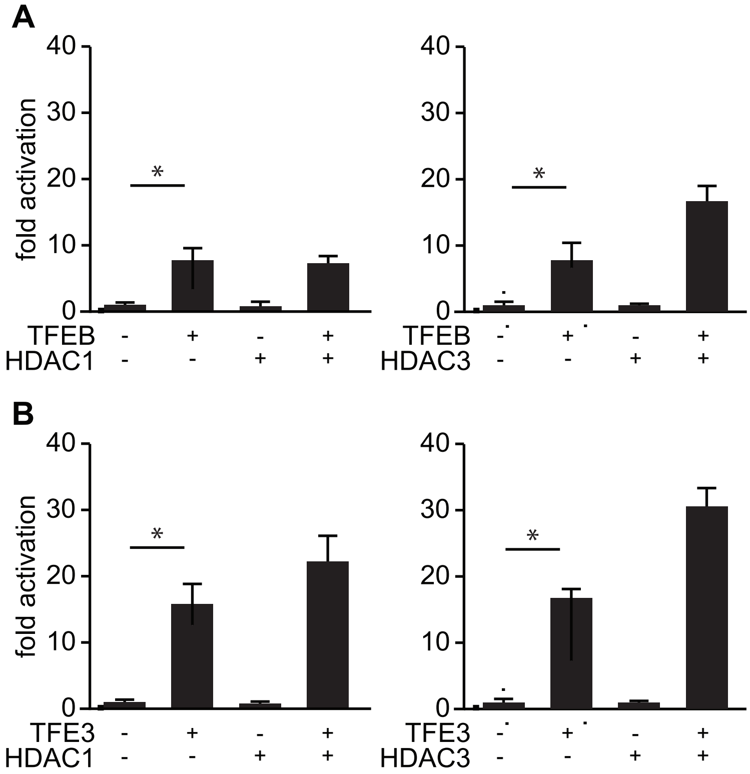

Supplementary Figure 1

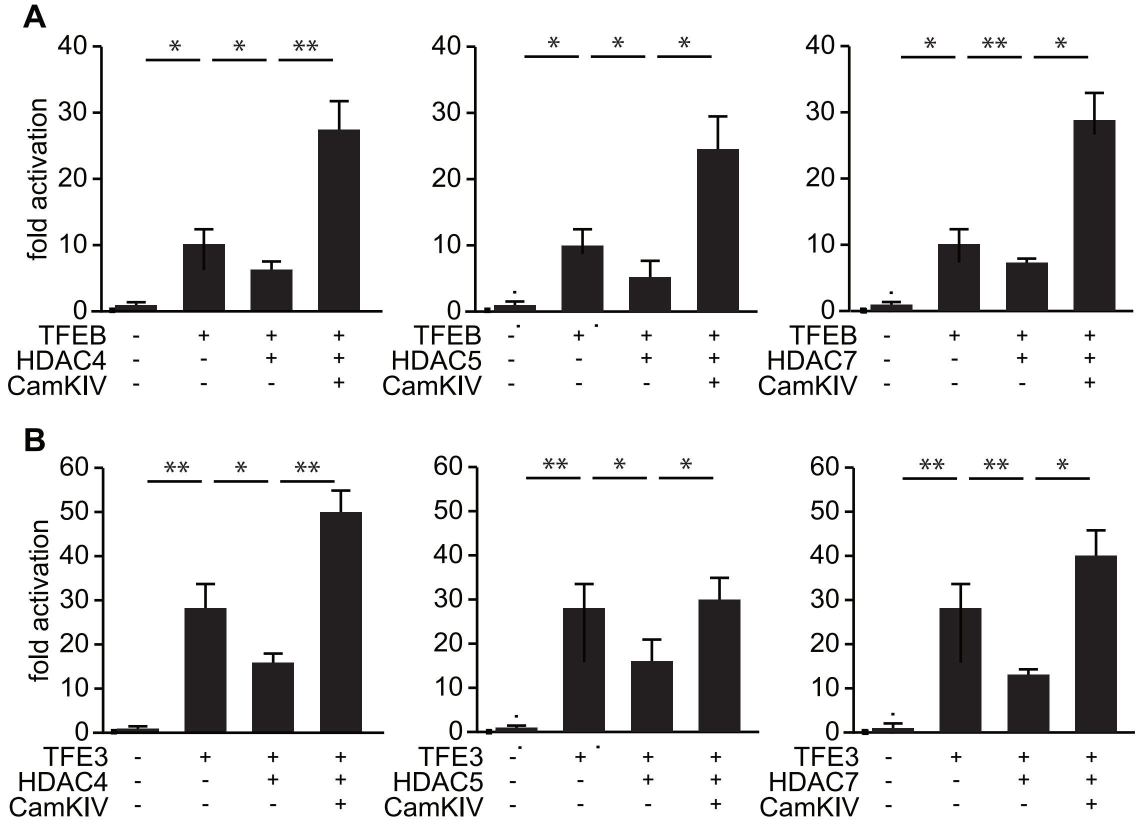

Supplementary Figure 2

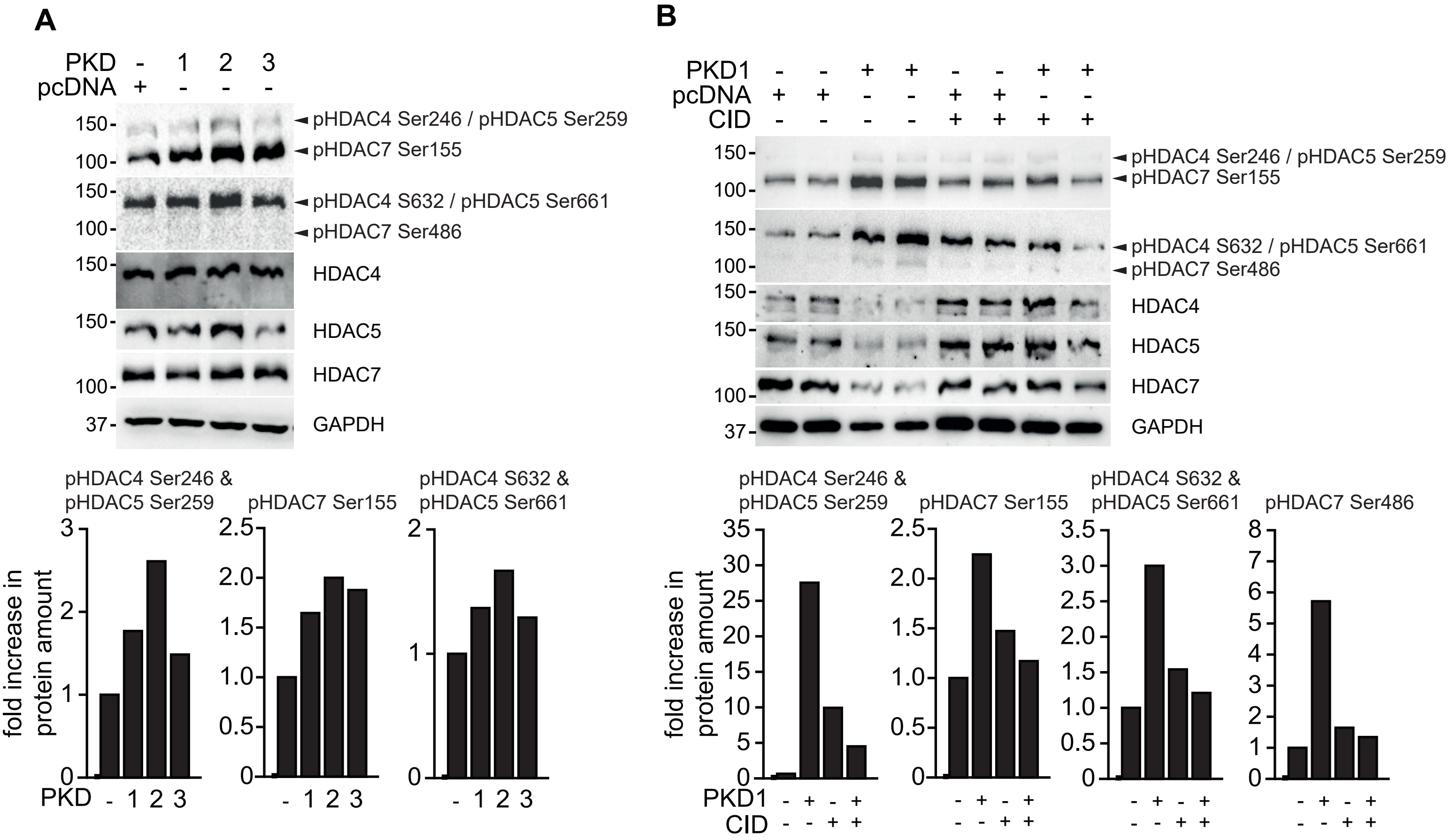

Supplementary Figure 3

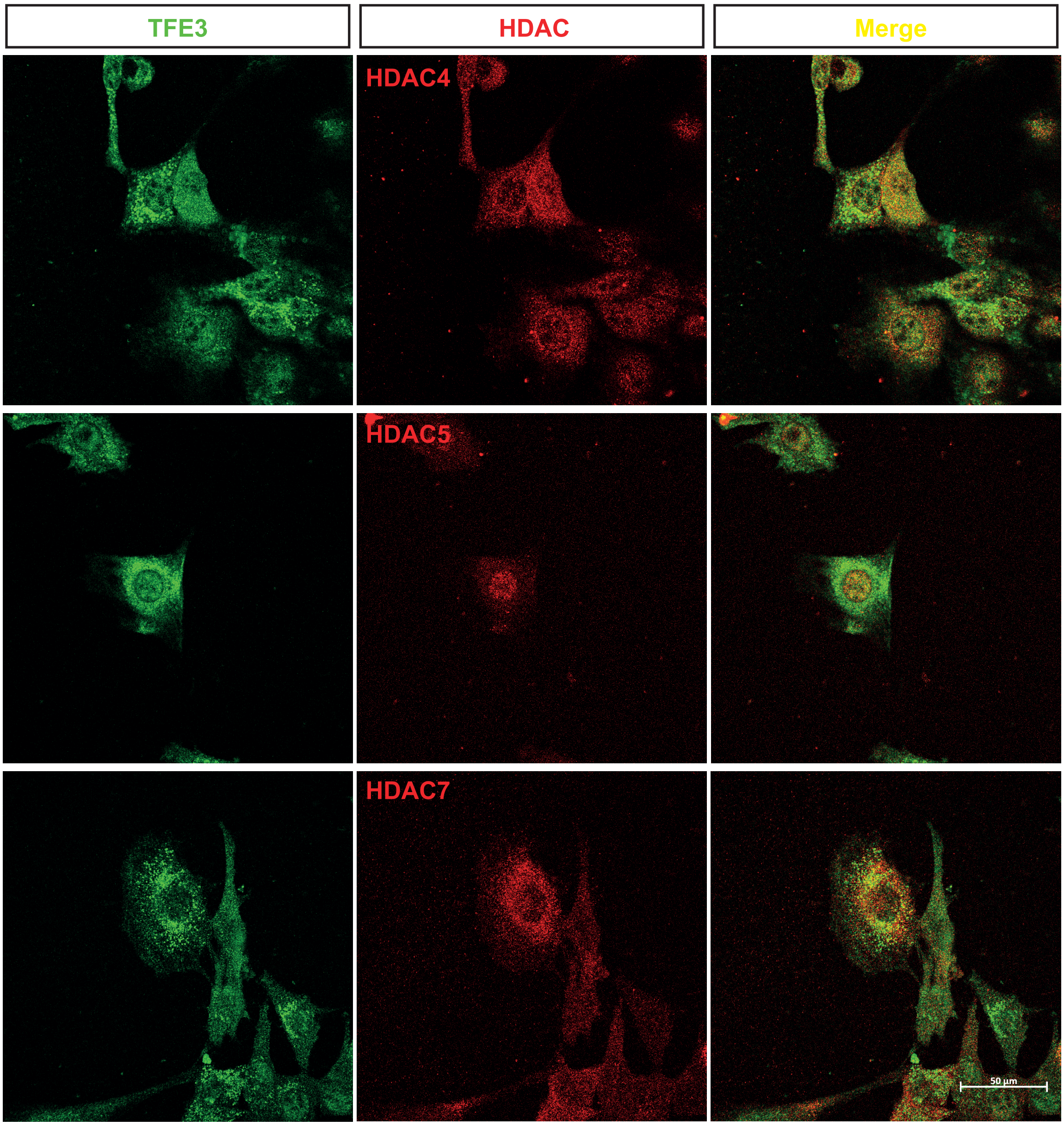

Supplementary Figure 4
